# An Innovative Decoupled Spatio-Vertical–Temporal Learning Model Based on Topography Guidance for Wind Vector Forecasting

**DOI:** 10.3390/s26196352

**Published:** 2026-10-08

**Authors:** Xinyu Liu, Huan Li, Xinyue Mo

**Affiliations:** School of Cyberspace Security/School of Cryptology, Hainan University, Renmin Avenue 58, Haikou 570228, China; 20233001523@hainanu.edu.cn (X.L.); lihuan@hainanu.edu.cn (H.L.)

**Keywords:** spatio-vertical–temporal decoupling, topography guidance mechanism, transformer, wind speed vector forecasting

## Abstract

**Highlights:**

**What are the main findings?**

- TG-SVTFormer improves multi-step wind speed forecasting by decoupling spatiotemporal features and preserving fine spatial details.
- Integrating topography and upper-air states adds physical constraints, reducing long-horizon prediction errors.

**What are the implications of the main findings?**

- Accurate fine-grained wind forecasts over complex terrain support secure grid integration and smart dispatching.
- The proposed framework offers a transparent, physics-aware methodology that researchers can adapt to mitigate the limited interpretability of other complex meteorological forecasting models.

**Abstract:**

Accurate wind speed forecasting (WSF) is pivotal for the secure grid integration of wind farms and the optimization of power dispatching. However, the stochastic nature of atmospheric dynamics and their complex interactions with local terrain pose significant challenges. Traditional spatiotemporal models inherently suffer from severe homogenization, irreversibly destroying fine-grained aerodynamic textures and leading to cumulative directional errors over extended horizons. To dismantle these limitations, this study proposes a novel Topography-Guided Spatio-Vertical–Temporal Transformer (TG-SVTFormer) for the multi-step forecasting of zonal and meridional wind vectors. Specifically, a dual-stage topography-guided mechanism explicitly injects static geographical variables (elevation, slope, and roughness) into both the spatial encoding and microscopic decoding phases, imposing key physical constraints on aerodynamic friction. Furthermore, the architecture employs a structured decoupled learning paradigm: a re-engineered Mix-Transformer spatial encoder deliberately eliminates deep-stage downsampling in favor of dilated convolutions—drastically expanding the global receptive field while preserving the intrinsic meteorological textures. Subsequently, the temporal evolution hub explicitly decouples the vertical dimension, utilizing upper-air atmospheric states to guide the target surface layer via simulated vertical momentum transport. The experiments showed the accuracy of TG-SVTFormer in predicting the wind speed with mean absolute error of 0.71 m/s in 3 h, 0.85 m/s in 6 h, and 1.06 m/s in 12 h. Compared to five existing baseline WSF models, the proposed TG-SVTFormer model demonstrates superior performance in wind speed forecasting and, more importantly, offers a transferable and physics-informed framework that can be adapted to other terrain-complex regions and extended to related renewable-energy and air-quality forecasting tasks.

## 1. Introduction

In recent decades, the excessive consumption of conventional fossil fuels, such as coal, oil, and natural gas, has significantly increased greenhouse gas emissions, exacerbating global climate change and triggering extreme weather events. To mitigate these environmental impacts and promote sustainable human growth, transitioning to alternative renewable energy is crucial. Wind energy, as a clean, inexhaustible, affordable, and ecologically friendly resource [1,2], has become a pivotal component in achieving carbon neutrality goals and creating a healthier global energy mix. Driven by its immense economic and environmental benefits, the global wind power industry has witnessed remarkable growth.

Despite these immense advantages, the large-scale integration of wind power is severely constrained by its inherent drawbacks. The stochasticity, intermittency, and extreme volatility of atmospheric conditions present a significant challenge to the secure and stable operation of power systems [3]. Such unpredictable fluctuations can restrict the responsiveness of control systems, lower economic efficiency, and even cause severe wind abandonment when wind power is extensively integrated into the grid [4]. Consequently, accurate and robust wind speed forecasting (WSF) is imperative for diminishing these impacts, reducing operational costs, ensuring economic dispatch, and maintaining the stable integration of large-scale wind farms into the power grid.

However, conventional WSF predominantly focuses on scalar wind speed prediction at isolated geographical points. With the ongoing advancement of wind energy, the spatial layout of modern wind farms is progressively expanding across extensive areas. Consequently, for pragmatic implementation, wind prediction models must be able to forecast the distribution of wind dynamics over an extensive spatial extent. This implies that traditional single-point or even multi-point scalar approaches are fundamentally inadequate for comprehensive wind power deployment. Instead, secure grid integration heavily relies on regional forecasting and the simultaneous prediction of both wind speed and wind direction (i.e., Wind Speed Vector Forecasting, WSVF) [5]. Therefore, developing a WSVF system that jointly predicts multi-step U (east–west component) and V (north–south component) wind components has emerged as a crucial frontier in meteorological and energy research [6].

Currently, wind speed prediction methodologies can be broadly categorized into three primary groups: physical approaches [7,8], statistical approaches [9], and artificial intelligence (AI)-based data-driven methods [10,11]. Physical methods, such as Numerical Weather Prediction (NWP) systems, commence with the fundamental principles of atmospheric thermodynamics and fluid dynamics [12]. These systems simulate atmospheric processes by solving massive, complex nonlinear partial differential equations [13,14]. Well-known physical models include the Integrated Forecasting System (IFS) from the European Centre for Medium-Range Weather Forecasts (ECMWF) and the Weather Research and Forecasting (WRF) model [15,16]. While these physical models boast exceptional interpretability and are effective for large-scale, long-term weather forecasting, they are plagued by an enormous computational resource overhead. Furthermore, these intricate models are notoriously rigid, highly sensitive to initial boundary conditions, and require excessively long execution times, rendering them ill-suited for the rapid, real-time responses demanded by short-term WSVF.

In sharp contrast to physical approaches, statistical methods carry a significantly lower computational burden and are easier to deploy. Statistical approaches primarily rely on historical sequence data to establish linear connections between past and future states. Classical techniques include time series models such as the autoregressive integrated moving average (ARIMA) [17], generalized autoregressive conditional heteroskedasticity models, and grey system forecasting methods [18,19]. Although these methods demonstrate efficiency in short-term predictions for stable environments, their reliance on linearity fundamentally restricts their capacity to capture the highly intricate, nonlinear volatility inherent in wind dynamics. When the forecast horizon extends, these linear assumptions lead to severe error accumulation, drastically widening the prediction gap during multi-step forecasting tasks [20].

To overcome the limitations of physical and statistical models, AI-based approaches have garnered unprecedented attention owing to their robust nonlinear fitting capabilities and flexibility [21]. Initially, researchers predominantly utilized traditional machine learning techniques, such as support vector machines (SVM) [22], random forests (RF) [23], and extreme learning machines (ELM) [24]. While these algorithms improved prediction accuracy over statistical methods, they fundamentally rely on laborious manual feature extraction and exhibit limited abstraction capabilities [25]. Consequently, deep learning models, serving as pioneers in the current era, have rapidly dominated the field. As foundational architectures in the evolution of deep learning, Deep Neural Networks (DNNs) and Multilayer Perceptrons (MLPs) mimic the synaptic mechanisms of biological nervous systems and have been extensively explored for wind speed forecasting [26]. However, escalating the structural depth of these models inevitably triggers a massive expansion in the parameter space, which not only complicates the optimization process but also increases the susceptibility to overfitting. Furthermore, their inherent structural limitations prevent them from effectively capturing bidirectional temporal correlations, thereby constraining their utility in rigorous sequence modeling. To alleviate this computational burden, Convolutional Neural Networks (CNNs) introduce a parameter-sharing paradigm to compress model complexity, while the integration of residual blocks [27] permits the training of substantially deeper networks. In applied contexts, CNN architectures have been successfully deployed for real-world predictions, including electricity consumption forecasting [28,29], and one-dimensional convolutions have been explicitly leveraged to forecast both wind speed and direction [30], demonstrating their robust feature extraction capabilities in time-series analysis. Despite these advantages, CNNs inherently struggle with the dynamic processing of variable-length sequential inputs, and the empirical optimization of convolutional kernel dimensions remains a non-trivial challenge. Conversely, Recurrent Neural Networks (RNNs) are fundamentally designed for sequence modeling; their cyclic topology dynamically processes inputs of varying lengths, circumventing the rigid requirement for fixed-length data while effectively capturing temporal dependencies. Although theoretically capable of learning long-range correlations, standard RNNs are frequently bottlenecked in practice by gradient vanishing and explosion phenomena, hindering their broader deployment. To address these specific vulnerabilities, advanced variants like Long Short-Term Memory (LSTM) networks and Gated Recurrent Units (GRUs) were engineered with sophisticated gating mechanisms that partially resolved the gradient vanishing issue. Ultimately, however, the strictly sequential nature of data processing along the time dimension in both LSTMs and GRUs fundamentally limits parallelization, potentially imposing severe bottlenecks on model training convergence and inference efficiency, especially for long sequences.

Recognizing that WSVF is inherently a highly complex spatiotemporal modeling problem, the focus of the research community has pivoted toward advanced spatiotemporal deep learning frameworks designed for multi-site joint forecasting. Convolutional Neural Networks (CNNs) and CNN-RNN hybrids (e.g., ConvLSTM) have been widely adopted to extract structural spatial patterns alongside temporal evolution. For instance, Joseph et al. [31] introduced an optimized CBiLSTM network that deeply mines temporal dependencies fused with convolutional features to address short-term wind forecasting. Similarly, Tian et al. [32] successfully employed a SmaAt-UNet architecture, leveraging the powerful spatial reconstruction capabilities of U-Net augmented with attention mechanisms to model complex, non-linear atmospheric structural patterns. However, CNN-based models are intrinsically limited by localized receptive fields, struggling to capture the long-range global dependencies of large-scale weather systems. To address this, Transformer models, which completely abandon sequential recurrence in favor of self-attention mechanisms, have revolutionized the field by offering a global view [33,34]. While the pioneering Vision Transformer (ViT) laid the foundation for global dependency modeling [35], hierarchical vision transformers, such as the Mix-Transformer (MiT) [36], have demonstrated remarkable prowess in extracting multi-scale spatial representations from meteorological grids. Concurrently, the positional-encoding-free design of Mix-Transformer inherently untethers the model from rigid input dimensions [36], offering crucial flexibility for handling meteorological grids with varying spatial resolutions. Furthermore, sophisticated attention-based hybrid models have been explicitly tailored for wind energy; for example, Lin et al. [37] developed GAOformer, which adaptively fuses spatiotemporal features by integrating a Graph Attention Network (GAT) with optimizable graph matrices to capture irregular spatial distributions.

From the broader perspective of energy meteorology and sustainable marine energy systems, recent cutting-edge studies further demonstrate the following. The WRF mesoscale model solves hydro-thermodynamic governing equations to realize high-resolution wind-field simulation over complex-terrain and coastal regions and is widely adopted as a physical tool for wind-resource assessment [38]. Deep learning frameworks based on LSTM and Transformer are capable of simultaneously correcting the systematic bias and temporal variability of ERA5 hourly wind speeds through a time-resolved scaling factor, thereby improving the accuracy of large-scale wind resource assessment [39]. To address the biases in macroscale meteorological grids caused by spatial smoothing of the relief, state-of-the-art existing studies have corrected ERA5 using the characteristic vertical scale of water vapor and the relative height differences among grid nodes, and have further refined reanalysis values with neural networks [40]. These refinement methods for ERA reanalysis values are still being developed worldwide. Frontier work oriented toward offshore energy systems has significantly improved the prediction accuracy of surge and mooring line tension responses for floating offshore wind turbines under extreme conditions such as typhoons, through cross-validation between CFD and high-fidelity tools as well as drag coefficient calibration [41]. Meanwhile, the design optimization of marine renewable energy devices such as tidal current turbines has widely adopted the integration of BEM, CFD, and experiments, so as to enhance the power output of shrouded turbines and reduce structural loads [42].

Despite the remarkable advancements achieved by the aforementioned state-of-the-art spatiotemporal models, a thorough review of the literature reveals two critical and fatal limitations that persistently hinder the accuracy of WSVF:

1. Topography Blindness: Wind flow in the lower atmospheric boundary layer is physically dictated by the aerodynamic friction and thermodynamic effects of the Earth’s surface. However, the vast majority of existing spatiotemporal models rely exclusively on historical meteorological variables, completely overlooking static topographical priors. They operate under a “topography blindness” paradigm, failing to recognize that geographic characteristics such as surface elevation, topological slope, and surface roughness drastically alter wind speed and direction, especially in complex mountainous or coastal terrains. Neglecting this vital geographical context severely severs the connection between the data-driven model and physical geographical reality, leading to catastrophic prediction failures in regions with abrupt terrain variations;
2. Entangled 4D Dynamics and the Neglect of Vertical Physics: Wind is the macroscopic manifestation of 4D atmospheric thermodynamics. Existing spatiotemporal models overwhelmingly treat this 4D system as an entangled “black box,” superficially coupling the spatial and temporal dimensions without modeling their distinct evolutionary mechanisms. More crucially, the majority of current research relies almost entirely on surface-level (10 m or 100 m) meteorological data, blatantly ignoring the fundamental physical principle of atmospheric circulation: surface wind vectors are predominantly driven by the vertical momentum transport from high-altitude atmospheric currents. By neglecting multi-pressure level data and failing to decouple the vertical dimension from the temporal evolution, existing models are mathematically incapable of capturing the true geostrophic forcing that governs boundary-layer wind vectors.

To systematically address the severe limitations of topography blindness and entangled 4D atmospheric dynamics, this paper proposes an innovative model specifically designed for multi-step WSVF, named topography-guided spatio-vertical–temporal transformer (TG-SVTFormer). The proposed model significantly differs from traditional approaches by bridging deep learning with atmospheric physical laws. The following innovations are presented in this study:

1. A novel dual-stage topography-guided mechanism is presented—the first to simultaneously inject smoothed topographic height, slope and topographic relief into spatial encoding and microscopic decoding. This provides physical constraints for perception of aerodynamic friction and terrain-induced wind shear.
2. Decoupled Spatio-Vertical–Temporal Learning, a two-stage paradigm that dismantles traditional spatiotemporal entanglement, is introduced. The encoder extracts pure multi-scale spatial features to prevent premature mixing, after which the vertical decoupled temporal hub captures temporal evolution.
3. The standard Mix-Transformer-B1 (MiT-B1) [36] backbone was customized for regional meteorological fields by replacing downsampling in Stages 3 and 4 with dilated convolutions. This design preserves fine-grained spatial textures while expanding the global receptive field to capture long-range atmospheric dependencies at full resolution.
4. Within the vertical decoupled temporal hub, TG-SVTFormer introduces High-Altitude Physics Guidance by leveraging upper-level atmospheric wind vectors to direct target-layer wind predictions. Moving beyond the traditional paradigm, this strategy mitigates error accumulation in long-horizon forecasting and enhances model robustness against chaotic meteorological volatility.

The remainder of this paper is organized as follows. Section 2 delineates the technical methodologies employed for the construction of the prediction model. Section 3 is dedicated to the evaluation of the experimental results, incorporating an in-depth comparative analysis between the proposed model and the benchmark models. Finally, Section 4 provides a conclusive summary of the study and suggests promising avenues for future research.

## 2. Materials and Methods

### 2.1. Dataset

Northern China and Mongolia are endowed with abundant wind energy resources and currently host expansive onshore wind power bases of a massive scale globally. Driven by the critical engineering necessity to optimize wind energy dispatch in these highly productive regions, the study area is specifically selected within the latitude and longitude range of [37.5° N–53.5° N, 95.5° E–127.5° E]. The highly heterogeneous terrain and complex local climate systems in this area make it an ideal testbed for evaluating topography-guided wind forecasting models. The wind velocity data for this region are sourced from the ERA5 reanalysis dataset provided by the ECMWF (Reading, United Kingdom). ERA5 offers a globally consistent format with regular spatial and temporal resolution, making it highly convenient for comparative analyses across different regions and time periods. Moreover, as one of the most authoritative and widely used reanalysis products, ERA5 has been extensively validated and applied in a large body of atmospheric and renewable energy research. The spatial resolution of the selected data is 0.25° × 0.25°, yielding a well-defined grid of 64 × 128 observation points. The temporal span encompasses four consecutive years from January 2021 to December 2024, with a sampling interval of 1 h. In pragmatic wind power generation contexts, the hub height of modern commercial wind turbines is typically situated around 100 m above the surface [43]. Therefore, the wind vectors at 1000 hPa are selected as the forecasting targets in the hub-height wind prediction study. Furthermore, while the ERA5 dataset provides three-dimensional atmospheric fields across 13 vertical pressure scales (ranging from 50 to 1000 hPa), high-altitude layers have negligible direct mechanical influence on near-surface wind patterns. To eliminate redundant meteorological noise and optimize computational efficiency, we selectively extract wind data from seven lower-to-middle tropospheric levels: 400, 500, 600, 700, 850, 925, and 1000 hPa. Wind dynamics within these specific layers dominate the planetary boundary layer interactions and strongly influence the downward vertical momentum transfer, thereby exerting a significant thermodynamic impact on the target 1000 hPa surface wind field.

**Figure 1:**
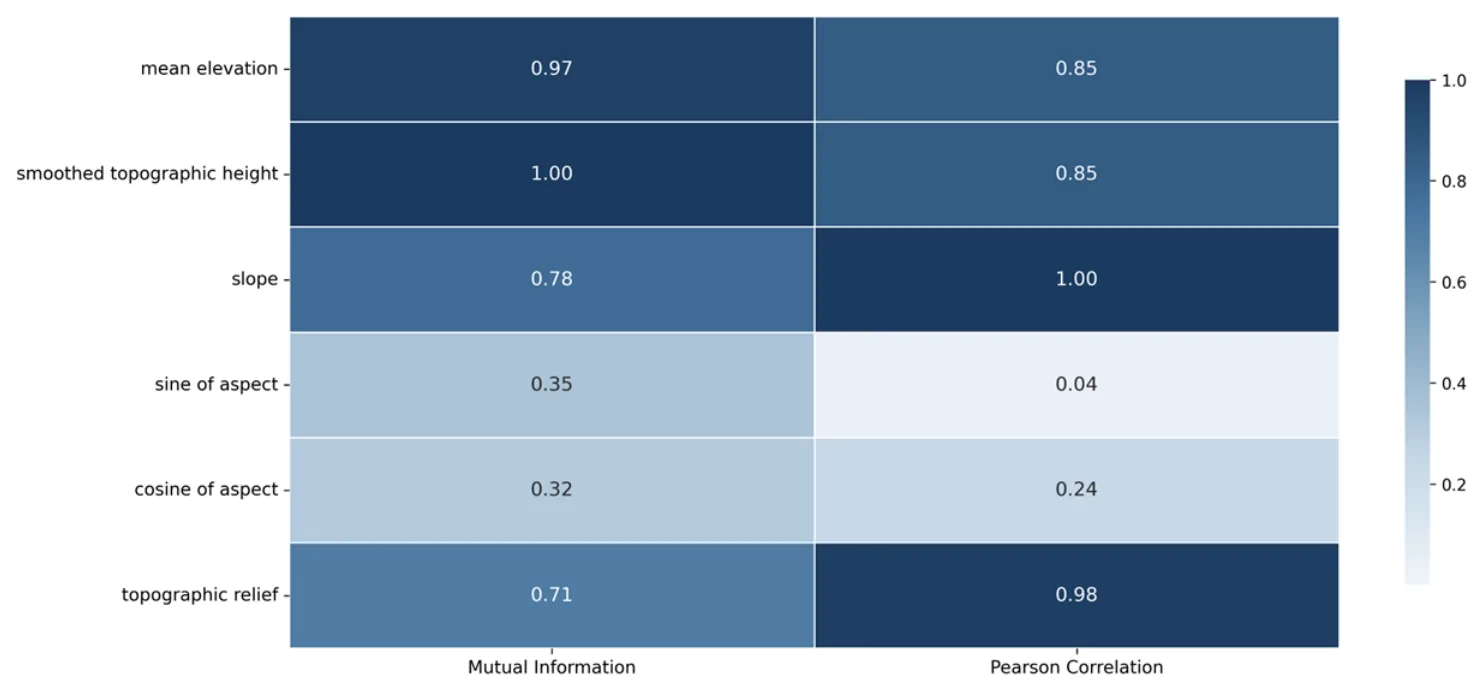
Statistical evaluation and selection of static topographical variables against mutual information and Pearson correlation coefficients.

To equip the model with crucial spatial physical constraints, high-resolution digital elevation model (DEM) data is acquired from the National Oceanic and Atmospheric Administration (NOAA). Initially, six topographical variables are calculated: mean elevation, smoothed topographic height, slope, sine of aspect, cosine of aspect, and topographic relief. As illustrated in Figure 1, to avoid feature redundancy, Mutual Information (MI) and Pearson Correlation are employed as a systematic feature selection criterion. Specifically, MI evaluates the statistical dependencies between candidate topographical features and the target wind field, while the Pearson Correlation Coefficient quantifies their direct linear association to eliminate redundant spatial noise and retain the most informative physical priors. Ultimately, three optimal auxiliary topographic variables are selected to guide the model, all of which are spatially downsampled to a 0.25° × 0.25° resolution to align with the meteorological tensors. Smoothed topographic height is extracted directly from the raw DEM data utilizing bilinear interpolation. Physically, it governs the macro-scale airflow trajectories, inducing significant large-scale blocking and channeling effects. Slope is mathematically derived based on the topographic gradient as:

(1) $$S=\sqrt{dx^{2}+dy^{2}}$$

where dx and dy denote the spatial gradients of the surface elevation along the longitudinal and latitudinal directions, respectively. It serves as an important boundary condition for characterizing localized wind shear and dynamic orographic lifting. Topographic relief is calculated using the standard deviation of elevation within a localized moving window, formulated as:

(2) $$T=\sqrt{E[X^{2}]-(E[X]{)}^{2}}$$

where X denotes the discrete surface elevation values within a specific localized moving window and E[⋅] signifies the mathematical expectation operator, which corresponds to the spatial mean over that localized window. This variable explicitly reflects the intensity of near-surface aerodynamic friction, which induces mechanical momentum loss and turbulence in the lower atmosphere. Notably, because the elevation data of the ocean area represent the depth of the seabed, we use the zero-conversion method to address the topographical data of the ocean area to avoid the influence of this on capturing the role of land topography [4].

### 2.2. Data Preprocessing

The primary research objective is formulated as a multi-step spatiotemporal forecasting task: predicting the future 12-h wind speeds based on the historical 12-h wind speed data, structurally guided by the three aforementioned static topographic matrices. To substantially improve training efficiency and numerical stability, the highly dynamic meteorological wind data is standardized using Z-Score Normalization to mitigate anomalies, whereas the static topographic variables are processed via Min-Max Normalization to preserve their spatial relative scales. To generate sequential samples, a sliding window technique is implemented with the number of sliding steps set to 3 h. Finally, the chronologically processed dataset is partitioned into a training set, a validation set, and a testing set with a ratio of 7:1.5:1.5, to prevent temporal data leakage. All data processing, model training and visualization were implemented using Python 3.10. The deep learning framework PyTorch 2.7 was used to build the proposed model.

### 2.3. Methods

**Figure 2:**
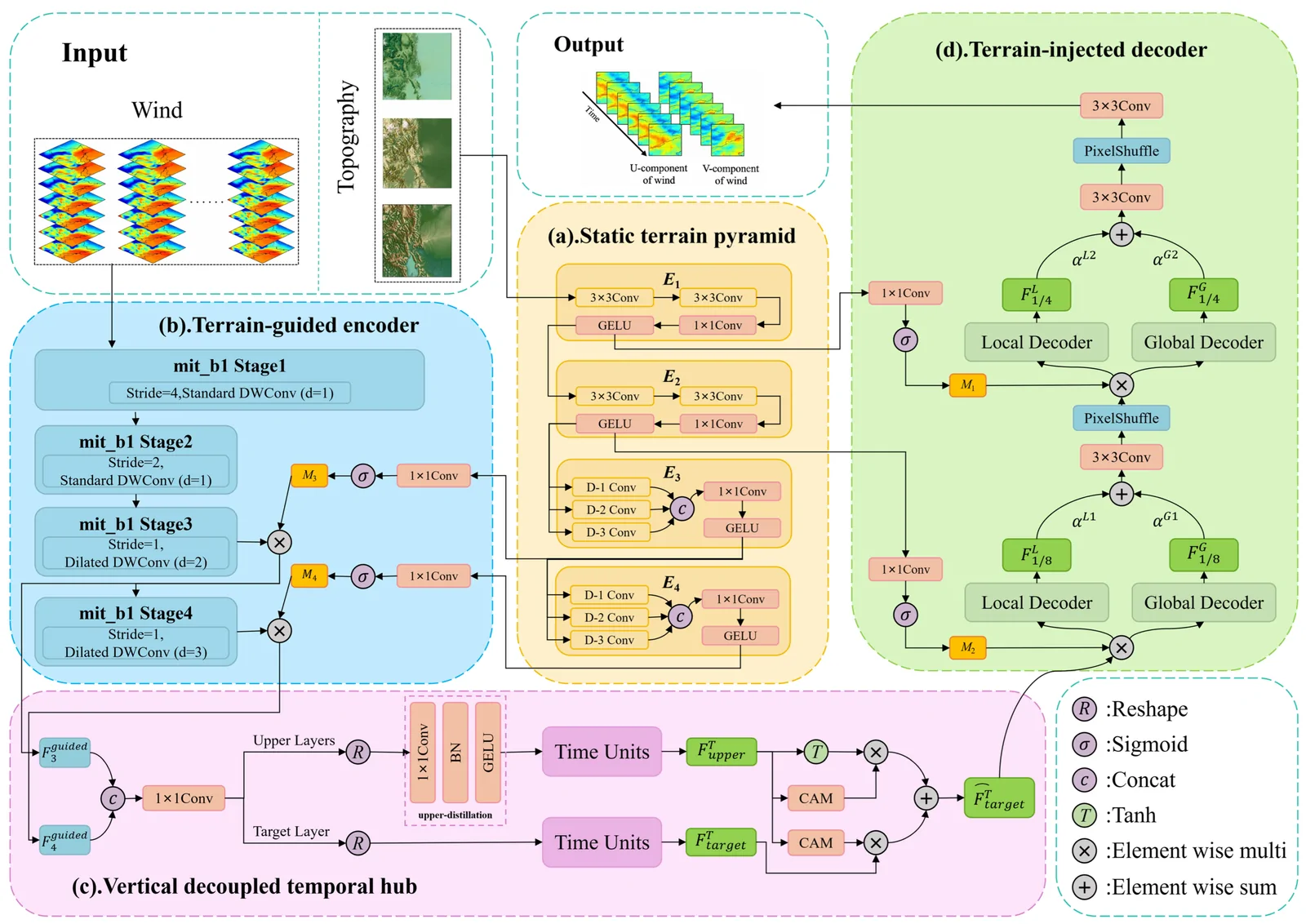
Framework of the proposed TG-SVTFormer model. (**a**): Static Terrain Pyramid, illustrating the model’s core terrain physical constraint module for extracting multi-scale static terrain prior information; (**b**): Terrain-Guided Encoder, presenting the full multi-stage spatial feature extraction pipeline; (**c**): Vertical Decoupled Temporal Hub, achieving temporal feature decoupling and establishing physical constraint correlations between vertical and temporal dimensions; (**d**): Terrain-Injected Decoder, conducting feature upsampling and final meteorological field reconstruction.

In this section, the proposed TG-SVTFormer for WSVF is comprehensively detailed. To facilitate a clear understanding of the overall framework, Figure 2 illustrates the conceptual architecture of the TG-SVTFormer model alongside its multi-source input and output data flows.

Initially, three distinct static topographical variables (smoothed topographic height, slope and topographic relief) are ingested into a static terrain pyramid to construct multi-scale geographical physical constraints. Subsequently, the historical meteorological data are fed into a terrain-guided encoder—constructed upon a modified MiT-B1 backbone—which explicitly injects macro-scale terrain priors to capture extensive multi-scale spatial dependencies. Following spatial encoding, the extracted deep features are transferred into a vertical decoupled temporal hub. Within this framework, vertical atmospheric dynamics are explicitly decoupled to model time evolution across isolated pressure levels. Driven by a physically inspired design, high-level atmospheric wind vectors are extracted to modulate the target-layer representations—emulating the vertical momentum transport from stable geostrophic winds. Finally, a terrain-injected decoder refines and up-samples the fused representations to jointly forecast the multi-step wind speed and direction (U and V components). The detailed mathematical formulations and theoretical mechanisms of these core modules are systematically introduced in the ensuing subsections.

#### 2.3.1. Static Terrain Pyramid

In multi-step WSVF, the near-surface atmospheric flow is strongly perturbed by geographical characteristics, leading to complex aerodynamic friction and terrain-induced wind shear. As shown in Figure 2a, to allow the proposed TG-SVTFormer to integrate these key physical constraints, the static terrain pyramid module is systematically designed to construct multi-scale geographical representations.

Let the raw static topographical variables (smoothed topographic height, slope and topographic relief) be mathematically denoted as the geographical tensor:

(3) $$X_{topo}\in R^{C_{topo}\times H\times W}$$

where $C_{topo}=3$ represents the distinct feature channels, and H and W denote the spatial height and width of the forecasting grid, respectively. To match the hierarchical processing stages of the spatial encoder, the static terrain pyramid extracts multi-scale physical priors through a cascade of specialized convolutional blocks.

Initially, the micro-roughness feature map at a 1/4 spatial scale, denoted as $E_{1}\in R^{C_{topo,1}\times \frac{H}{4}\times \frac{W}{4}}$, is extracted to capture local aerodynamic surface resistance through two consecutive spatial downsampling layers:

(4) $$E_{1}=\sigma \left({Conv}_{1\times 1}\left({Conv}_{3\times 3,s=2}\left({Conv}_{3\times 3,s=2}\left(X_{topo}\right)\right)\right)\right)$$

where ${Conv}_{k\times k,s}$ denotes a 2D convolution operation with a kernel size of k and stride s, and σ(⋅) represents the Gaussian Error Linear Unit (GELU) activation function. Subsequently, the local slope feature map at a 1/8 spatial scale, denoted as $E_{2}\in R^{C_{topo,2}\times \frac{H}{8}\times \frac{W}{8}}$, is generated to reflect the physical wind shear caused by topographic undulations:

(5) $$E_{2}=\sigma \left({Conv}_{1\times 1}\left({Conv}_{3\times 3,s=2}\left({Conv}_{3\times 3,s=2}\left(E_{1}\right)\right)\right)\right)$$

To capture the extensive, multi-scale spatial dependencies during dispatch while minimizing further spatial information loss, a parallel dilated convolution architecture is deployed at the 1/8 locked scale to extract the macro-elevation feature map, denoted as $E_{3}\in R^{C_{topo,3}\times \frac{H}{8}\times \frac{W}{8}}$. By integrating parallel branches with varying dilation rates d∈{1,2,3}, the model significantly expands its geographical receptive field. The features from these branches are concatenated along the channel dimension and fused via a 1×1 convolution:

(6) $$E_{3}=\sigma \left({Conv}_{1\times 1}\left({Concat}_{d\in \{1,2,3\}}\left[{DConv}_{3\times 3,d}\left(E_{2}\right)\right]\right)\right)$$

where ${DConv}_{3\times 3,d}$ indicates a 3×3 dilated convolution with a dilation rate of d, and Concat denotes the channel-wise concatenation.

Following a similar parallel dilated architecture, a deeper geographical feature map, denoted as $E_{4}\in R^{C_{topo,4}\times \frac{H}{8}\times \frac{W}{8}}$, is further extracted to capture broader macro-scale terrain impacts. This stage utilizes parallel branches with dilation rates d∈{1,3,4}, which are likewise concatenated and fused:

(7) $$E_{4}=\sigma \left({Conv}_{1\times 1}\left({Concat}_{d\in \{1,3,4\}}\left[{DConv}_{3\times 3,d}\left(E_{3}\right)\right]\right)\right)$$

These hierarchical terrain priors ($E_{1},E_{2},E_{3},E_{4}$) establish a comprehensive geographical physical constraint system, which will subsequently be injected into both the spatial encoding and microscopic decoding phases.

#### 2.3.2. Terrain-Guided Encoder

The Transformer architecture, based on the self-attention mechanism [33], has demonstrated advantages in sequence modeling tasks in recent years. Compared to traditional Recurrent Neural Networks (RNN) or LSTMs, Transformers can process entire input sequences in parallel, thereby improving training efficiency and capturing long-range dependencies [43]. Building upon these advantages, the Mix-Transformer (MIT) paradigm has demonstrated strong feature extraction capabilities in dense prediction tasks due to its hierarchical architecture and overlapping patch embedding (OPE) mechanisms [36]. By progressively shrinking the spatial resolution of feature maps, the standard MIT backbone constructs multi-scale receptive fields, facilitating the extraction of both local and global contextual dependencies. Within this architectural family, the MiT-B1 variant is adopted as the foundational baseline. The rationale is rooted in the trade-off between representational capacity and computational overhead (as quantitatively summarized in Table 1). While the lightweight B0 variant lacks sufficient channel depth to encode complex meteorological thermodynamics, heavier variants (e.g., B2–B5) incur memory bottlenecks during the processing of multi-step 3D atmospheric tensors. Consequently, MiT-B1 strikes an optimal Pareto balance: it yields adequate embedding dimensions (scaling up to 512 channels) to map macro-scale aerodynamic boundaries, while maintaining an efficient parameter space. As shown in Figure 2b, the terrain-guided encoder is used for spatial feature extraction, which is an improved Mix-Transformer-B1 encoder with its original segmentation decoder head discarded.

To execute the proposed decoupled spatio-vertical–temporal paradigm, a structural data reshaping is performed prior to the spatial encoding phase. The raw dynamic atmospheric input is initially formulated as a high-dimensional tensor $X_{wind}\in R^{B\times T\times P\times 2\times H\times W}$, encompassing the batch size, historical time steps, vertical pressure levels, wind variables (U and V components), height, and width. To isolate horizontal aerodynamic textures from temporal and vertical entanglements, the temporal dimension T and pressure dimension P are folded into the batch dimension B. This yields a 2D-equivalent flattened tensor $X_{wind}^{flat}\in R^{(B\cdot T\cdot P)\times 2\times H\times W}$. By feeding this restructured tensor into the encoder, the network performs pure spatial feature extraction. This enables each specific time step and pressure level to be processed independently as a standalone spatial plane, establishing a decoupled spatial foundation for subsequent vertical–temporal evolution modeling.

However, directly deploying this standard MiT-B1 backbone to regional WSVF poses a physical dilemma. The conventional MIT architecture downsamples the spatial resolution by a factor of 32 (i.e., 1/4→1/8→1/16→1/32) across its four stages. In meteorological forecasting, this downsampling destroys fine-grained spatial flow patterns, leading to the homogenization of local aerodynamic textures and the loss of mesoscale wind shear boundaries.

To circumvent this limitation, a customized Topography-Guided Spatial Encoder is engineered to preserve spatial granularity. The core modification targets the OPE mechanism inherent in the MIT architecture. In standard configurations, Stage 3 and Stage 4 employ 2D convolutional projections with a stride of 2 to halve the spatial dimensions. To truncate this progressive downsampling, the architectural parameters of the OPE modules in the final two stages are reprogrammed. Specifically, the convolutional strides are overridden from s=2 to s=1, accompanied by symmetrically adaptive zero-padding (p=1) to ensure pixel-to-pixel receptive sliding without dimensional shrinkage. Let the atmospheric feature mapped to the stage l of the encoder be denoted as $X_{in}\in R^{(B\cdot T\cdot P)\times C_{l-1}\times H_{l-1}\times W_{l-1}}$. By enforcing this non-strided projection, spatial compression is aborted, yielding an intermediate feature map $X_{patch}\in R^{(B\cdot T\cdot P)\times C_{l}\times H_{l}\times W_{l}}$, where $H_{l}=H_{l-1}$ and $W_{l}=W_{l-1}$. Consequently, the progressive spatial shrinkage is halted at Stage 2, locking the spatial resolution of the dynamic wind field at a 1/8 scale for the remainder of the encoding process. This targeted truncation averts the severe loss of micro-meteorological features, providing a topological foundation for downstream prediction.

While locking the resolution preserves local details, it causes a deficit in the global receptive field. To compensate for this without introducing additional computational overhead, dilated depth-wise convolutions are integrated into the Transformer’s feed-forward network (FFN). The standard Multilayer Perceptron (MLP) is upgraded to dynamically process variable-frequency wind field textures. The feature transformation within the Transformer block is formulated as:

(8) $${\tilde{X}}_{attn}=Attention(LayerNorm(X_{patch}))+X_{patch}$$

(9) $$\begin{array}{c} X_{FFN}=\\ Dropout(Linear(Dropout(GELU({Conv}_{d}^{DW}(Linear(LayerNorm({\tilde{X}}_{attn}))))))) \end{array}$$

(10) $$X_{out}=X_{FFN}+{\tilde{X}}_{attn}$$

where $DWConv_{d}$ denotes a dilated depth-wise convolution and the dilation rate d is set to 2 in Stage 3 and 3 in Stage 4. This dilated architecture effectively breaks the bottleneck of localized receptive fields, broadening the spatial perception of distant wind corridors and allowing the model to capture mesoscale weather system variations without compromising feature map dimensions.

From a physical standpoint, relying solely on atmospheric data is insufficient for modeling near-surface aerodynamics. To constrain the dynamic atmospheric features using the underlying geographical reality, a vertically aware terrain injection mechanism is executed at the end of Stage 3 and Stage 4. This selective late-stage modulation is designed to align with the principles of aerodynamic scale-matching. In shallow stages (Stage 1 and Stage 2), the network primarily extracts high-frequency, micro-scale atmospheric fluctuations; injecting macro-scale topographical constraints here would provoke semantic misalignment and overwhelm delicate local gradients. In contrast, by Stage 3 and Stage 4, the dilated Transformer blocks have constructed an expansive receptive field capable of encapsulating broader mesoscale weather systems and macro wind corridors. Modulating these deep thermodynamic representations with static terrain mimics the macroscopic physical reality where large-scale geographical boundaries govern macro-aerodynamic flows. Given the static topography feature $E_{l}\in R^{(B\cdot T)\times C_{topo,l}\times H_{l}\times W_{l}}$ extracted from the terrain pyramid, a cross-vertical gating tensor $M_{l}^{1}$ is generated through a 1×1 convolutional mapping. Since the terrain is fundamentally two-dimensional while the wind field features span P vertical pressure levels, the network expands the terrain’s influence:

(11) $$M_{l}^{1}=Conv_{1\times 1}(E_{l})\in R^{(B\cdot T)\times (P\cdot C_{l})\times H_{l}\times W_{l}}$$

where $Conv_{1\times 1}$ denotes a 1×1 convolution. Subsequently, $M_{l}^{1}$ is reshaped along the pressure dimension to geometrically align with the 3D atmospheric tensor, yielding $M_{l}^{2}\in R^{(B\cdot T\cdot P)\times C_{l}\times H_{l}\times W_{l}}$. The output feature $F_{l}$ of the spatial encoder is then modulated via an element-wise sigmoid gating operation:

(12) $$F_{l}^{guided}=F_{l}⊙\sigma (M_{l}^{2})$$

where $F_{l}\in R^{(B\cdot T\cdot P)\times C_{l}\times H_{l}\times W_{l}}$ is the normalized feature tensor from the Transformer blocks, ⊙ denotes element-wise multiplication and σ is the Sigmoid activation function. Ultimately, this P-dimensional expanded topographic gating mechanism propagates surface roughness and elevation boundaries onto all atmospheric pressure layers, encouraging the encoded representations to better reflect real-world aerodynamic principles.

#### 2.3.3. Vertical Decoupled Temporal Hub

Following the spatial encoding phase, the network acquires multiscale deep spatial representations. However, atmospheric flows are governed by vertical stratification and continuous temporal evolution. As shown in Figure 2c, to circumvent the traditional spatiotemporal entanglement, we propose the vertical decoupled temporal hub. This module initially dismantles the feature tensors along the vertical axis, independently isolating the aerodynamic states of both the target prediction layer and the upper-air levels. Subsequently, it tracks their temporal evolutionary trajectories in parallel and leverages the upper-air dynamics to regulate the target-layer representations, physically aligning with the inter-layer atmospheric interactions.

Initially, the deep feature maps $F_{l}^{guided}(l\in \{3,4\})$ from Stage 3 and Stage 4 are concatenated and processed by a 1×1 convolutional fusion layer. The shallow representations from Stage 1 and Stage 2 are deliberately bypassed; these early stages primarily extract fine-grained, localized textures that are heavily contaminated by micro-scale atmospheric noise and lack the expansive receptive field necessary for macro-flow modeling. In contrast, Stage 3 and Stage 4 encapsulate broad mesoscale weather systems, providing the optimal macroscopic context for vertical dynamic analysis. This fusion operation compresses the multi-scale channels into a unified embedding dimension $c_{emb}=256$, generating the base tensor $F_{emb}$. To construct the vertical physical constraints, $F_{emb}$ is mathematically decoupled along the atmospheric pressure axis. The features belonging to the target prediction layer are isolated as $F_{target}$, while the remaining feature tensors from the higher pressure levels are ingested into an upper-distillation layer. This layer—comprising a 1×1 convolution, Batch Normalization, and GELU activation—aggregates the multidimensional high-altitude states into a consolidated upper-air representation $F_{uppper}$.

**Figure 3:**
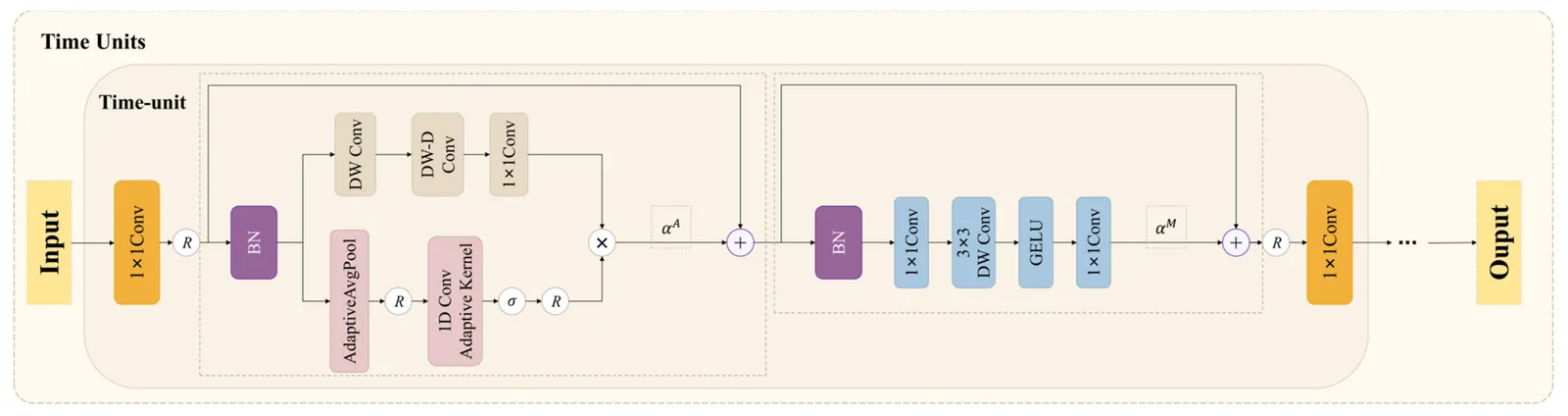
Structure of the Time Units.

Subsequently, to capture long-range temporal dependencies without corrupting the previously learned spatial structures, $F_{target}$ and $F_{upper}$ are fed into two parallel and independent time units [4]. Figure 3 shows the structure of the time units. To execute this robust temporal projection, each Time Unit is constructed as a hierarchical sequence of 4 cascaded time-unit modules. To prevent computational bottlenecks when processing the high-dimensional spatiotemporal tensor, each time-unit employs an adaptive bottleneck design. The input tensor is adaptively mapped into a layer-specific core channel dimension through a conditional 1×1 convolution for pre-reduction only when dimensional compression is necessitated, processed by the core computational engine, and subsequently expanded to the target dimension through another conditional 1×1 convolution for post-expansion exclusively when channel upsampling is dictated.

At its core, to capture the short-time aerodynamic fluctuations and long-time trends of the wind field, the time-unit adopts a residual dual-phase architecture to decouple intra-frame spatial extraction and inter-frame temporal evolution. Let $Z\in R^{B\times T\cdot C_{core}\times H\times W}$ denote the input tensor to the time-unit sub-block (TUSubBlock), where $C_{core}$ represents the dimension pre-reduced by a 1×1 convolution.

In the first phase, the normalized tensor is bifurcated into two parallel pathways. To extract the intra-frame spatiotemporal evolution patterns, the Spatial–Temporal branch employs a cascade of a 3×3 depthwise convolution and a dilated depthwise convolution. This specific combination effectively expands the sensory field, obtaining a receptive field comparable to that of a large kernel convolution without incurring substantial computational overhead. A subsequent 1×1 convolution integrates these cross-channel features, yielding the spatial representation $Z^{1}$:

(13) $$Z^{1}=Conv_{1\times 1}\left(Conv_{DW-D}\left({Conv}_{DW}\left(BN(Z)\right)\right)\right)$$

where BN represents Batch Normalization, $Conv_{DW-D}$ denotes a depth-wise dilated convolution, and ${Conv}_{DW}$ denotes a depth-wise convolution. Concurrently, to explicitly regulate the inter-frame relationship of the historical sequence, the temporal gating branch captures long-range temporal dependencies. An Adaptive Average Pooling operation first collapses the spatial dimensions, generating $Z_{GAP}\in R^{B\times (T\cdot c_{core})\times 1\times 1}$. To explicitly expose the pure time axis, this tensor is mathematically reshaped and permuted into $Z_{time}\in R^{(B\cdot c_{core})\times 1\times T}$. A 1D convolution with an adaptive kernel size slides across this isolated temporal axis to capture sequential evolutionary patterns. This is followed by a Sigmoid activation to output the continuous temporal gating weights $Z^{2}$:

(14) $$Z^{2}=Fold\left(\sigma \left(Conv_{1d}\left(Unfold\left(AAP(BN(Z))\right)\right)\right)\right)$$

where $Conv_{1d}$ denotes a one-dimensional convolution with convolution kernel d, and AAP denotes adaptive average pooling. Afterward, the two branches of information are fused. The temporal gating weights dynamically modulate the intra-frame spatial features:

(15) $$Z_{attn}=Z+\alpha^{A}\left(Z^{1}⊙Z^{2}\right)$$

where $\alpha^{A}$ is the corresponding learnable scale for the residual.

In the second phase, a Mix-MLP module performs non-linear cross-channel transformations to refine the evolved representations. This phase incorporates a 3×3 depth-wise convolution sandwiched between two 1×1 convolutions and a GELU activation function. The final output is mathematically expressed as:

(16) $$Z_{out}=Z_{attn}+\alpha^{M}\left(Conv_{1\times 1}\left(\delta \left(Conv_{3\times 3}^{DW}\left(Conv_{1\times 1}(BN(Z_{attn}))\right)\right)\right)\right)$$

where δ denotes a GELU activation function and $\alpha^{M}$ is the corresponding learnable scale for the Mix-MLP residual.

After the evolutionary process of multiple cascaded time-units, the final block directly projects the temporal sequence length. By configuring the output channel dimension of the final expansion layer, the network maps the feature space from $\left(T\cdot c_{emb}\right)$ to $\left(T_{pred}\cdot c_{emb}\right)$. The resulting tensor is structurally reconstructed and restored to $R^{(B\cdot T_{pred})\times c_{emb}\times H\times W}$, achieving the multi-step direct prediction from historical sequences to future spatiotemporal states.

**Figure 4:**
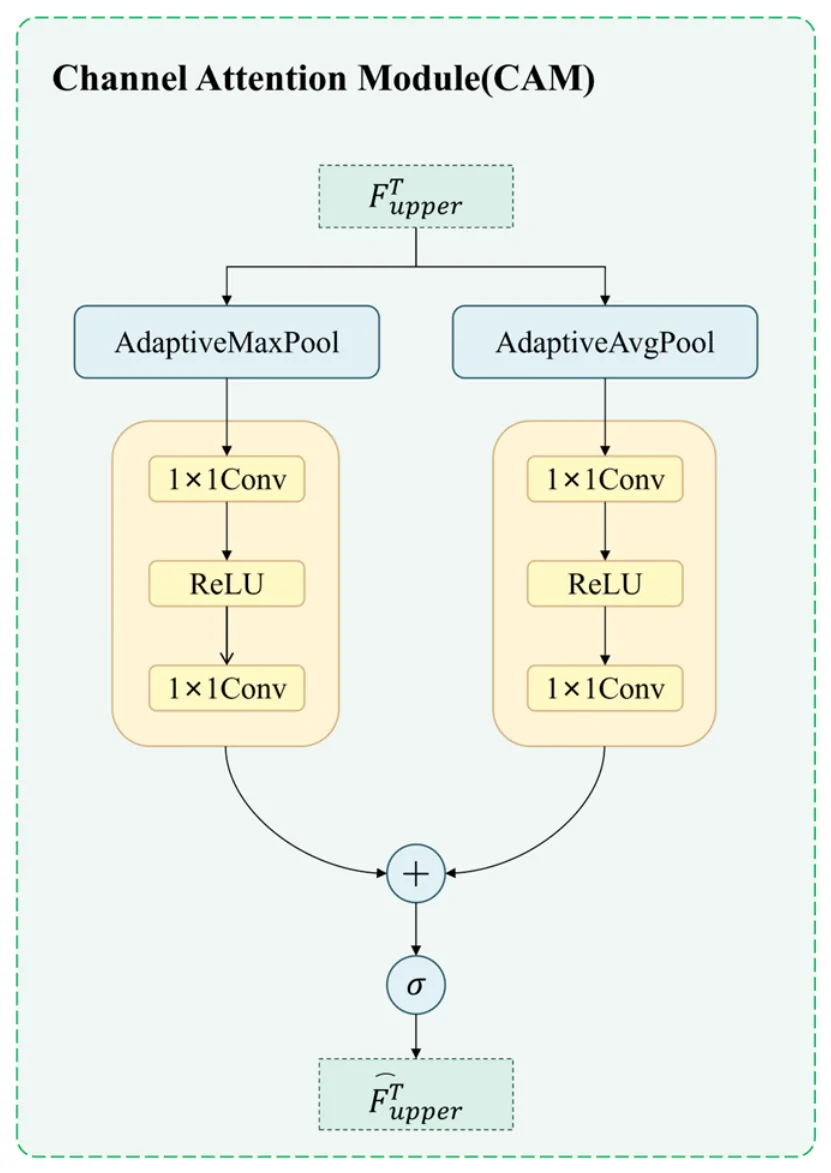
Structure of the Channel Attention Mechanism (CAM).

Ultimately, to physically emulate the vertical momentum transport and inspired by the gating mechanisms of Long Short-Term Memory (LSTM) networks, the independent temporal outputs from the target layer $F_{target}^{T}$ and the upper-air layer ($F_{upper}^{T}$) are integrated through a dynamic vertical guidance mechanism. Specifically, the upper-air state $F_{upper}^{T}$ is processed by two parallel Channel Attention Mechanisms (CAM) to generate adaptive modulation weights, whose network structure is shown in Figure 4. Internally, each CAM compresses the spatial dimensions via concurrent adaptive maximum and average pooling, followed by a shared Multi-Layer Perceptron (MLP), consisting of two 1×1 convolutional weights and a ReLU activation, to capture cross-channel dependencies. The resulting outputs are element-wise summed and activated by a Sigmoid function to produce the attention maps:

(17) $$F_{amp}=Conv_{1\times 1}\left(\phi \left(Conv_{1\times 1}(AMP(F))\right)\right)$$

(18) $$F_{aap}=Conv_{1\times 1}\left(\phi \left(Conv_{1\times 1}(AAP(F))\right)\right)$$

(19) $$CAM(F)=\sigma \left(F_{amp}+F_{aap}\right)$$

where  ϕ denotes a ReLU activation function and AMP denotes adaptive max pooling. These weights act as specialized gates to regulate the fusion of atmospheric layers:

(20) $${\hat{F}}_{target}^{T}=F_{target}^{T}⊙CAM^{1}(F_{upper}^{T})+CAM^{2}(F_{up}^{T})⊙\mathrm{Tanh}(F_{upper}^{T})$$

where Tanh denotes the tanh activation function. In this formulation, $CAM^{1}$ operates analogously to a forget gate, filtering redundant or noisy target-layer features, while $CAM^{2}$ coupled with the tanh activation acts as an input gate, injecting highly relevant high-altitude momentum variations into the surface prediction. Modulating the target-layer dynamics with upper-air temporal states via this LSTM-inspired gating mechanism encourages the target-layer representations to better reflect vertical aerodynamic interactions, thereby improving the continuous temporal tracking of the wind vector field.

#### 2.3.4. Terrain-Injected Decoder

The final phase of the TG-SVTFormer framework aims to reconstruct the macroscopic fluid representations back into regional wind vectors. Standard decoders typically rely on interpolation or transposed convolutions, which tend to smooth out fine-grained aerodynamic boundaries. As shown in Figure 2d, to circumvent this over-smoothing dilemma, a progressive multi-scale terrain gating and dual-branch collaborative decoding paradigm is formulated. This architecture explicitly injects microscopic geographical constraints ($E_{1}$ and $E_{2}$) into the upsampling trajectory. At the 1/8 spatial scale, the future atmospheric representation ${\hat{F}}_{target}^{T}$ extracted from the evolution hub encounters the local slope prior $E_{2}$. Specifically, employing the physically inspired terrain gating mechanism introduced in Section 2.3.2, $E_{2}$ is projected into a spatial mask $M_{2}$ via a 1×1 convolution coupled with a Sigmoid activation. This mask modulates the fluid feature via element-wise multiplication, guiding the network to perceive terrain-induced wind shear. The terrain-perceptive tensor ${\hat{F}}_{1/8}$ is mathematically expressed as:

(21) $$M_{2}=\sigma ({Conv}_{1\times 1}(E_{2}))$$

(22) $$F_{1/8}={\hat{F}}_{target}^{T}⊙M_{2}$$

**Figure 5:**
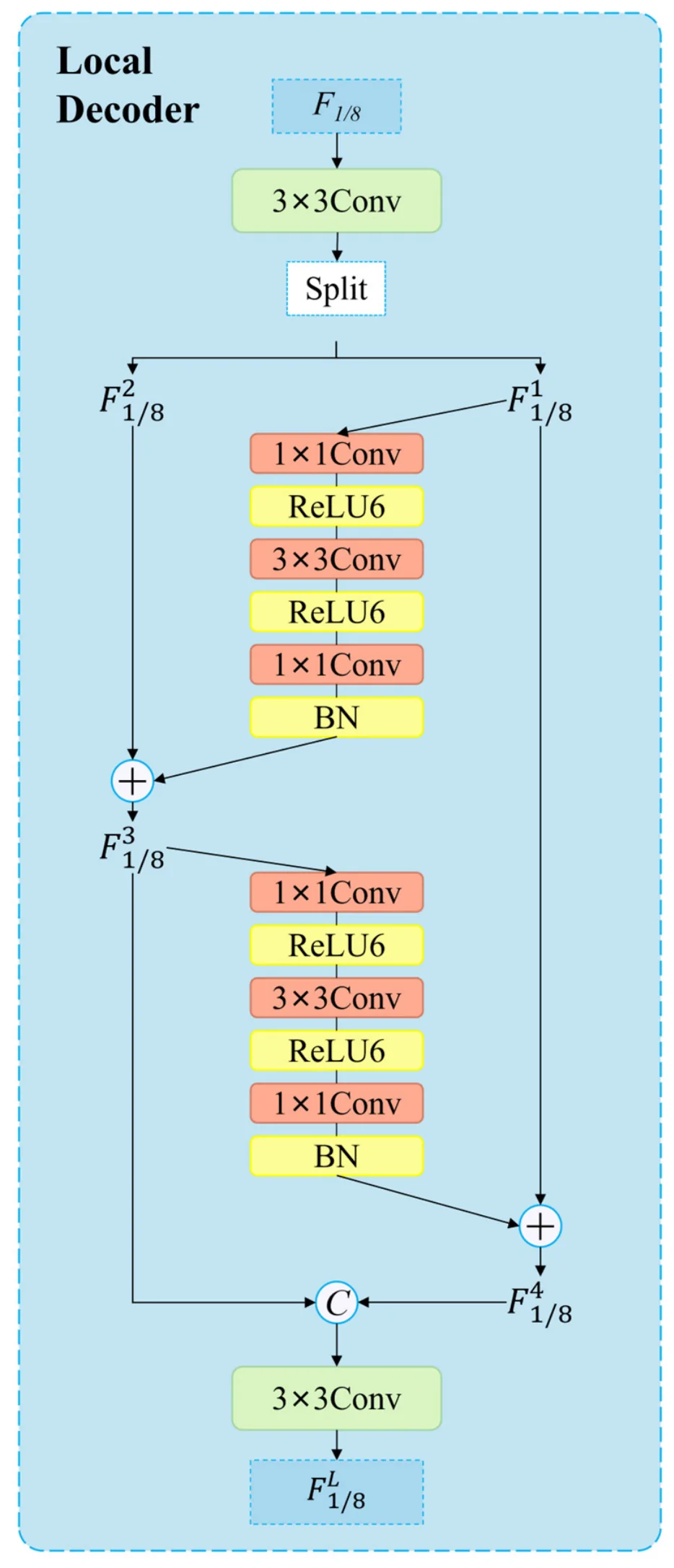
Structure of the Local Decoder.

**Figure 6:**
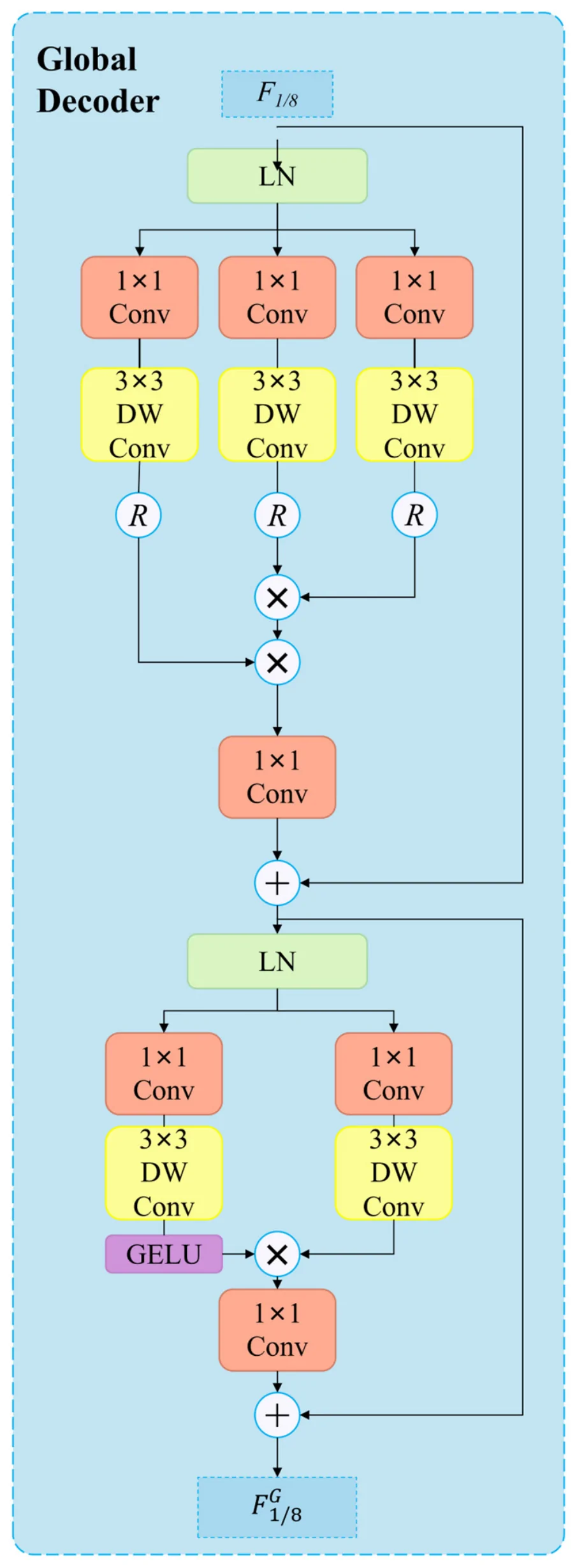
Structure of the Global Decoder.

To reconstruct the deep fluid representations back into original physical space while resolving the semantic misalignment between localized high-frequency aerodynamic turbulence and global continuous flow structures, $F_{1/8}$ is ingested into a parallel dual-branch decoding architecture—comprising a Local Decoder and a Global Decoder—whose detailed network structure is illustrated in Figure 5 and Figure 6. The evolution of the wind field inherently encapsulates complex micro-scale fluctuations, such as sharp wind shear. Traditional convolutional operations inevitably discard critical high-frequency components during the spatial upsampling process, which is detrimental to capturing acute meteorological dynamics. To circumvent this, the Local Decoder is architected upon an Invertible Neural Network (INN) [4,44]. The INN topology facilitates interactive information transfer while rigorously preserving feature integrity, allowing high-frequency localized aerodynamic textures to be retained as a foundation for the subsequent sub-pixel reconstruction. The terrain-perceptive tensor $F_{1/8}$ is initially mapped via a 3×3 convolution and subsequently partitioned into two independent sub-tensors, $F_{1/8}^{1}$ and $F_{1/8}^{2}$. To preserve the high-frequency wind shear boundaries, an alternating conditional update mechanism is executed, where the transformation can be expressed as:

(23) $$F_{1/8}^{1},F_{1/8}^{2}=Split(Conv_{3\times 3}(F_{1/8}))$$

(24) $$F_{1/8}^{3}=F_{1/8}^{2}+W_{inn}(F_{1/8}^{1})$$

(25) $$F_{1/8}^{4}=F_{1/8}^{1}+W_{inn}(F_{1/8}^{3})$$

(26) $$F_{1/8}^{L}=Conv_{3\times 3}(Concat[F_{1/8}^{3};F_{1/8}^{4}])$$

where $W_{inn}$ denotes the inverse convolution intermediate convolution process, which sequentially comprises a 1×1 expansion convolution, a 3×3 spatial convolution, and a 1×1 projection convolution.

Physically, the wind field is influenced by local climatological factors and regulated by large-scale meteorological systems. Large-scale meteorological forcings, such as monsoons, dictate the overall kinetic patterns and long-term evolutionary trends of the atmosphere. Capturing these global topological patterns and achieving an organic integration of localized features with macroscopic flow structures are important for reflecting the spatiotemporal dynamics of wind speed. The self-attention mechanism possesses a significant advantage in extracting global patterns by directly modeling long-range dependencies across arbitrary spatial locations. Simultaneously, to balance computational efficiency and reconstruction performance, a lite transformer block [4,45] is employed as the fundamental unit of this global decoding branch. The Q, K and V representations are independently generated via three parallel mapping sequences. Each sequence comprises a 1×1 convolution cascaded with a 3×3 depth-wise convolution (DWConv), which can be expressed as follows:

(27) $$Q=Conv_{3\times 3}^{DW^{q}}\left(Conv_{1\times 1}^{q}(LN{(F}_{1/8}))\right)$$

(28) $$K=Conv_{3\times 3}^{DW^{k}}\left(Conv_{1\times 1}^{k}(LN(F_{1/8}))\right)$$

(29) $$V=Conv_{3\times 3}^{DW^{v}}\left(Conv_{1\times 1}^{v}(LN(F_{1/8}))\right)$$

where LN represents Layer Normalization. Next, we dimensionally transform Q, K, and V to obtain $\hat{Q}\in R^{\left(B\cdot T_{pred})\times c_{emb}\times (H\cdot W\right)}$, $\hat{K}\in R^{\left(B\cdot T_{pred})\times c_{emb}\times (H\cdot W\right)}$, and $\hat{V}\in R^{\left(B\cdot T_{pred})\times c_{emb}\times (H\cdot W\right)}$. The attention computation process can be summarized as follows:

(30) $$F_{attn}=Conv_{1\times 1}(Softmax(\hat{Q}\times {\hat{K}}^{T}/\tau )\cdot \hat{V})+F_{1/8}$$

where Softmax denotes the SoftMax activation function. Here, τ is a learnable scaling parameter used to control the size of the dot product of K and Q before applying the SoftMax function. Afterward, feature transformation is performed via a regular feedforward network, which can be represented as follows:

(31) $${F_{attn}}^{\prime }=GELU\left(W_{1\times 1}^{a}{W_{3\times 3}^{DW}}^{a}\left(LN(F_{attn})\right)\right)⊙W_{1\times 1}^{b}{W_{3\times 3}^{DW}}^{b}\left(LN(F_{attn})\right)$$

(32) $$F_{1/8}^{G}=Conv_{1\times 1}({F_{attn}}^{\prime })+F_{attn}$$

where GELU denotes the GELU activation function. Two learnable parameters are introduced to balance the global spatial features and local spatial features adaptively:

(33) $${\hat{F}}_{1/8}=\alpha^{L1}\cdot F_{1/8}^{L}+\alpha^{G1}{\cdot F}_{1/8}^{G}$$

where $\alpha^{L1}$ and $\alpha^{G1}$ denote local and global weighting factors, respectively.

Following the feature refinement, a sub-pixel convolution mechanism expands the spatial resolution while mitigating the checkerboard artifacts inherent in conventional upsampling. The refined tensor ${\hat{F}}_{1/8}$ is upsampled to the 1/4 scale, yielding $F_{1/4}$:

(34) $$F_{1/4}={PS}_{2}(Conv_{3\times 3}({\hat{F}}_{1/8}))$$

where ${PS}_{2}$ denotes twofold sub-pixel expansion.

To prevent structural redundancy, this exact terrain gating and dual-branch decoding paradigm is recursively applied at the 1/4 scale. The micro-roughness prior $E_{1}$ modulates $F_{1/4}$, which then undergoes dual-branch decoding to synthesize the final refined state ${\hat{F}}_{1/4}$. Ultimately, a fourfold sub-pixel expansion paired with a terminal linear projection reconstructs the deep tensor into the target zonal and meridional wind vectors $Y_{pred}$:

(35) $$Y_{pred}={Conv}_{3\times 3}\left({PS}_{4}\left({Conv}_{3\times 3}\left({\hat{F}}_{1/4}\right)\right)\right)$$

where ${PS}_{4}$ denotes fourfold sub-pixel expansion.

#### 2.3.5. Physics-Constrained Hybrid Loss Function

Regional WSVF inherently requires the precise reconstruction of both the numerical magnitude and the complex topological morphology of the wind field. Traditional deep learning approaches predominantly rely on the Mean Squared Error (MSE) as the sole optimization objective. However, optimizing solely based on a global MSE is often inadequate for WSVF tasks. First, MSE tends to excessively penalize large numerical outliers while ignoring local structural coherence, resulting in overly smoothed prediction maps that can blur fine-grained meteorological textures (e.g., wind shear boundaries and cyclonic vortices). Second, conventional MSE computes the U and V components independently, neglecting the physical vector nature of wind and its periodic directional mutations. To address these limitations, a novel physics-constrained hybrid loss function is employed. This mechanism integrates global numerical regression, spatial topological preservation, and wind direction physical constraints.

To guarantee the macroscopic numerical fidelity of the predicted wind speeds, the global MSE is retained as the foundational optimization objective. For the ground truth tensor y and the predicted tensor $\hat{y}$, this base global loss is defined mathematically as the Frobenius norm of their difference:

(36) $$L_{Glo}=\|y-\hat{y}{\|}_{F}^{2}$$

where y denotes the true wind speed and $\hat{y}$ denotes the predicted wind speed. To explicitly capture the spatial skeleton and dynamic mutation boundaries of the wind field, a Spatial Structural Similarity (SSIM) loss is introduced. Unlike standard pixel-wise distance metrics, SSIM encourages the network to perceive local statistics—specifically the mean (μ), variance ($\sigma^{2}$), and covariance ($\sigma_{pt}$)—within a sliding window across the spatial domain. By optimizing the structural similarity, the model is guided to reconstruct the high-frequency topological dependencies of atmospheric flows rather than merely memorizing absolute grid values. The spatial structure loss is expressed as:

(37) $$L_{SSIM}=1-SSIM(\hat{y},y)$$

where SSIM(⋅) denotes the SSIM calculation. Furthermore, addressing the unique challenges of the WSVF task requires an explicit physical constraint on the wind direction. Physically, the wind direction at any given grid point is determined by the decomposition of the wind speed vector. Assuming that the U and V wind components at an observation point are defined as $U_{g}$ and $V_{g}$, the wind direction is dynamically deduced as follows:

(38) $$\Theta =\arctan2(V_{g},U_{g})$$

The true and predicted wind directions can be expressed as follows:

(39) $$\Theta =\arctan2(Y_{v},Y_{u}),\;\hat{\Theta }=\arctan2({\hat{Y}}_{v},{\hat{Y}}_{u})$$

Crucially, wind direction is a continuous cyclic variable defined over a 2π(360°) period. In this physical context, the true angular difference between 359° and 1° is merely 2°, yet a naive arithmetic subtraction yields an erroneous 358°. To adhere to this physical periodic boundary condition, a truncated angular difference ΔΘ is implemented. This conditional truncation encourages the model to consistently target the shortest physical angular distance:

(40) $$L_{Ang}=\left\{\begin{array}{c} |\hat{\Theta }-\Theta |,|\hat{\Theta }-\Theta |\leq \pi \\ 2\pi -|\hat{\Theta }-\Theta |,|\hat{\Theta }-\Theta |>\pi \end{array}\right.$$

Finally, these three distinct modules are harmoniously fused into a comprehensive optimization target. The total physics-constrained hybrid loss $L_{Total}$ is defined as:

(41) $$L_{Total}=\omega L_{Glo}+(1-\omega )(L_{SSIM}+L_{Ang})$$

where ω acts as the balancing hyperparameter to weigh the global numerical constraints against the sum of the high-frequency spatial topological loss and the vector direction loss. This physics-guided fusion mechanism helps suppress physically inconsistent wind field predictions and promotes stable multi-objective optimization convergence during training.

## 3. Results

### 3.1. Evaluation Metrics

To systematically evaluate the predictive performance of the proposed model on regional wind fields, four metrics are employed: Root Mean Square Error (RMSE), Mean Absolute Error (MAE), Anomaly Correlation Coefficient (ACC), and Wind Direction Forecasting Accuracy (WDFA). Considering the Earth’s spherical geometry, standard pixel-wise spatial averaging introduces systematic spherical deformation errors. To account for the latitudinal variation in grid cell area, a latitude weighting factor, L(j), is applied:

(42) $$L(j)=\frac{\cos(lat(j))}{\frac{1}{N_{lat}}\sum_{j}^{N_{lat}}\cos\left(lat(j)\right)}$$

where lat(j) represents the specific latitude at the j-th index, and $N_{lat}$ is the total number of latitudinal grid points.

Leveraging this physics-informed weighting, RMSE and MAE quantify the absolute deviation between predicted and observed wind speed components (calculated independently for U and V winds):

(43) $$RMSE=\frac{1}{N_{predictions}}\sum_{i}^{N_{predictions}}\sqrt{\frac{1}{N_{lat}N_{lon}}\sum_{j}^{N_{lat}}\sum_{k}^{N_{lon}}L(j){\left({\hat{Y}}_{i,j,k}-Y_{i,j,k}\right)}^{2}}$$

(44) $$MAE=\frac{1}{N_{predictions}}\sum_{i}^{N_{predictions}}\frac{1}{N_{lat}N_{lon}}\sum_{j}^{N_{lat}}\sum_{k}^{N_{lon}}L(j)\left|{\hat{Y}}_{i,j,k}-Y_{i,j,k}\right|$$

where $N_{predictions}$ denotes the predicted time length, $N_{lat}$ and $N_{lon}$ denote the number of grid points in the latitudinal and longitudinal directions, i, j, and k denote the time, width, and height indices, Y  denotes the true value, and $\hat{y}$ denotes the predicted value.

Furthermore, to assess spatial topological consistency, the ACC evaluates the correlation between forecast anomalies and actual anomalies relative to the historical climatology ξ. Here the climatology is defined as $\xi y_{h,w}=\frac{1}{N_{time}}\sum y_{h,w}$ and $N_{time}$ denotes the length of all the times to be predicted:

(45) $$ACC=\frac{\sum_{i,j,k}^{N}L(j)({\hat{Y}}_{i,j,k}-\xi )(Y_{i,j,k}-\xi )}{\sqrt{\sum_{i,j,k}^{N}L(j)({\hat{Y}}_{i,j,k}-\xi {)}^{2}\sum_{i,j,k}^{N}L(j)(Y_{i,j,k}-\xi {)}^{2}}}$$

Furthermore, to specifically evaluate wind direction forecasting, we employ the $WDFA_{\alpha }$ metric. Across the H×W spatial grid, this metric quantifies the proportion of points where the directional prediction error falls below a predefined angular threshold α (typically set to $90^{\circ }$, $45^{\circ }$, and 22.5°). The formulation of $WDFA_{\alpha }$ is given by:

(46) $${WDFA}_{\alpha }=\frac{Count(diff(pred,true)<\alpha )}{H\times W}\times 100\%$$

(47) $$diff(pred,true)=\left\{\begin{array}{c} |pred-true|,|pred-true|\leq 180\\ 360-|pred-true|,|pred-true|>180 \end{array}\right.$$

where Count(⋅) denotes counting the number of eligible observations. For example, for 64 × 128 grid points in Northeast China, if 7700 grid points have an angular difference of less than 22.5 degrees, then $WDFA_{\alpha =22.5}=\frac{7700}{64\times 128}\times 100\%=93.99\%$.

### 3.2. Ablation Experiments

To verify the effectiveness of each core module in the proposed TG-SVTFormer, a systematic set of ablation experiments was conducted. Using twelve-hour historical wind speed data, predictions are made for the U and V wind components for the next twelve hours. The prediction results are shown in Table 2, Table 3, Table 4 and Table 5. Four model variants, referred to as C1 to C4, are compared: the model with the terrain-guided encoder removed (C1), the model with the vertical decoupled temporal hub removed (C2), the model with the terrain-injected decoder removed (C3) and the complete TG-SVTFormer (C4).

The experimental results demonstrate that the complete TG-SVTFormer model exhibits significant advantages over all its ablated variants across all forecast horizons and evaluation metrics. Specifically, model C1 shows a marked decline in ACC and WDFA, highlighting the critical role of the terrain-guided encoder in capturing extensive, multi-scale spatial dependencies. Without the initial injection of static topographical variables, C1 struggles to accurately perceive aerodynamic friction and terrain-induced wind shear, leading to suboptimal spatial representations. Similarly, model C2 suffers the most severe performance degradation, particularly in long-term forecasting. This substantial error accumulation underscores the indispensability of the vertical decoupled temporal hub. Due to entangling spatial and temporal dynamics into a conventional “black box” and failing to leverage high-level atmospheric wind vectors to physically guide target-layer predictions, C2 exhibits exceedingly poor robustness against chaotic meteorological volatility over extended horizons. Furthermore, compared with the complete architecture, model C3 exhibits consistently inferior performance, particularly in the highly strict WDFA (α=22.5°) metric. This indicates that while the encoder captures macroscopic features, the terrain-injected decoder plays a crucial role in the microscopic reconstruction phase. The explicit re-injection of topographical variables at the decoding stage provides key physical constraints that help mitigate the degradation of fine-grained spatial textures, enabling the network to better reconstruct localized wind field topologies. Ultimately, the proposed integrated model, TG-SVTFormer, achieves optimal performance across all evaluation metrics, fully demonstrating the synergistic effects of each physics-guided component within the deep decoupled architecture.

### 3.3. Model Evaluation

**Figure 7:**
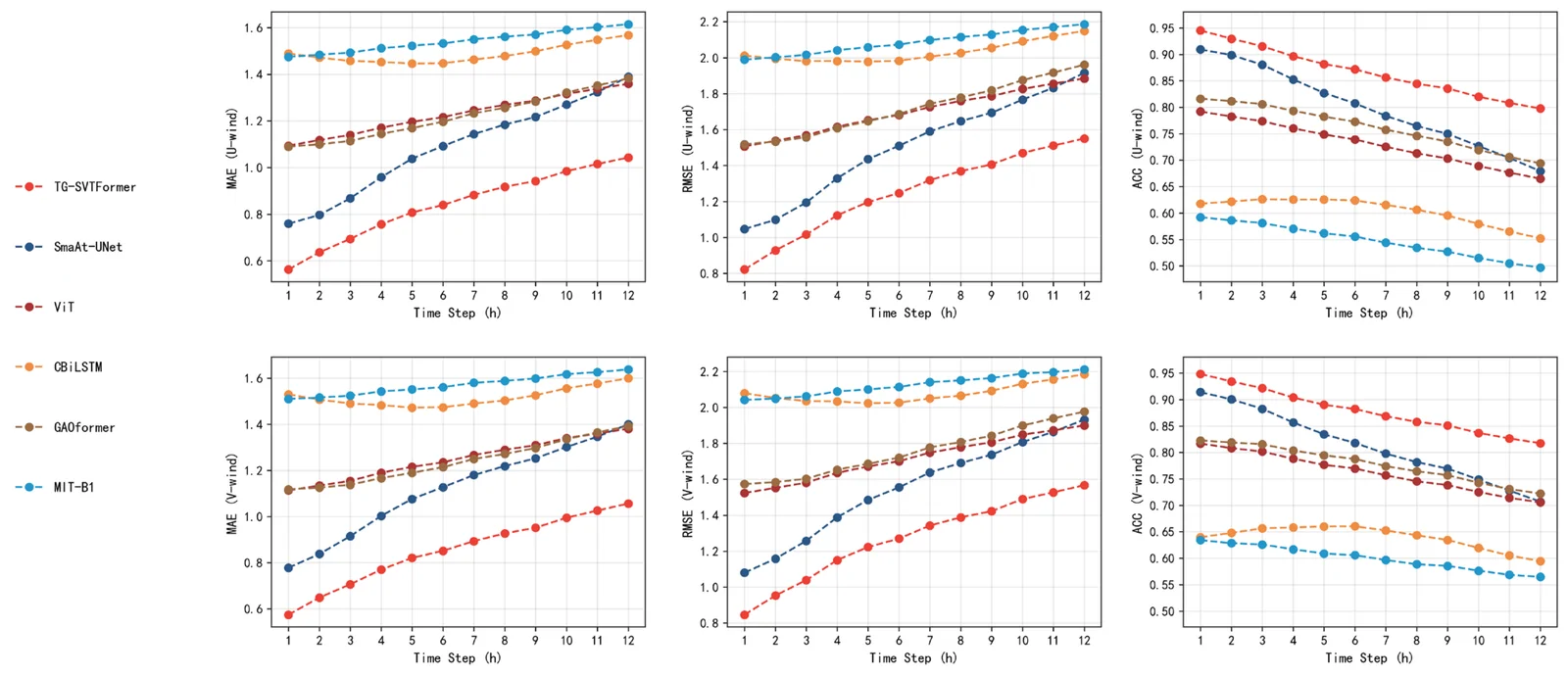
Prediction comparison of U-wind and V-wind of WSV with different models.

To comprehensively evaluate the predictive capability of the proposed TG-SVTFormer in regional WSVF, it was compared with five baseline models: ViT [35], MiT-B1 [36], CBiLSTM [31], GAOformer [37], and SmaAt-UNet [32]. The evaluation encompasses continuous forecasting horizons ranging from 1 to 12 h. The results of the RMSE, MAE, ACC and WDFA assessments of the wind speed prediction for each method are listed in Table 6, Table 7, Table 8 and Table 9. The experimental results (Figure 7) clearly show that the proposed TG-SVTFormer consistently outperforms all competing baselines across all forecasting horizons. For example, at the 1-h horizon, TG-SVTFormer achieves an RMSE of 0.85 m/s for the V-wind, reducing the error by 21.3% compared to the second-best model SmaAt-UNet (1.08 m/s) and by 59.1% against the traditional CBiLSTM model (2.08 m/s). This consistent superiority extends up to the 12-h horizon (t + 12), where TG-SVTFormer maintains an RMSE of 1.57 m/s (V-wind) and 1.55 m/s (U-wind), demonstrating its long-term stability. Similar superior trends are observed in the MAE metric, indicating the model’s robustness against extreme wind speed outliers.

TG-SVTFormer addresses key limitations of existing baseline architectures through three core innovations: first, unlike RNN-based models (e.g., CBiLSTM) whose sequential processing causes gradient vanishing, cumulative multi-step errors, and performance degradation over extended 12-h horizons due to implicit spatial topology modeling, TG-SVTFormer uses a parallelized temporal evolution mechanism to partially circumvent error accumulation; second, whereas Vision and Spatial-Attention models (e.g., ViT, MiT-B1, and SmaAt-UNet) lack explicit geographical awareness, leading to considerable topography-blind biases in highly non-stationary wind fields (despite SmaAt-UNet’s competitive short-term results), whereas TG-SVTFormer introduces a Dual-Stage Topography-Guided Mechanism that injects elevation, slope, and roughness directly into both spatial encoding and microscopic decoding phases to perceive aerodynamic friction and terrain-induced wind shear; third, spatiotemporal models like GAOformer show relatively weaker performance due to a entanglement of spatial and temporal features. For instance, GAOformer achieves a 6-h U-wind RMSE of 1.69 m/s, whereas TG-SVTFormer achieves 1.25 m/s. By contrast, TG-SVTFormer uses a Decoupled Spatio-Vertical–Temporal Learning framework that leverages multi-pressure-level UV wind data to physically guide target surface levels with upper-level atmospheric features, thereby filtering out near-surface chaotic noise, capturing deterministic synoptic-scale patterns, and contributing to strong long-term prediction robustness.

In regional power grid dispatching, predicting scalar magnitude alone is insufficient; accurately forecasting the wind flow spatial distribution and direction is equally critical. Therefore, ACC was utilized to evaluate the correlation between forecast anomalies and actual anomalies relative to historical climatology, and WDFA was employed to validate vector directional accuracy. As illustrated in Table 9, the WDFA metric—evaluated under varying angular tolerance thresholds ($\alpha =90^{\circ },45^{\circ },22.5{}^{\circ}$)—serves as a key indicator of the model’s robustness in multivariate coupling. Figure 7 visually illustrates that TG-SVTFormer establishes a clear lead across all thresholds and time steps. Under the strictest tolerance threshold (α=22.5°) at 1 h, TG-SVTFormer achieves an accuracy of 80.16%, vastly outperforming SmaAt-UNet (61.94%) and GAOformer (68.78%). More importantly, as the spatial correlations of the wind field evolve rapidly over extended horizons, traditional models experience a substantial degradation in directional accuracy due to cumulative spatiotemporal errors. For instance, at the 12-h mark (α=22.5°), CBiLSTM and MiT-B1 decline to an unacceptable 34.33% and 34.79%, respectively. In stark contrast, TG-SVTFormer retains a WDFA of 57.88% and consistently achieves the highest ACC. This spatial and directional fidelity demonstrates that by decoupling the vertical and temporal dimensions and integrating topographic aerodynamic constraints, TG-SVTFormer effectively captures the physical evolution trajectory of the wind vector field. For short-term turbine yaw adjustments and medium-term dispatching arrangements, TG-SVTFormer provides a reliable aerodynamic reference.

### 3.4. Spatiotemporal Visualization and Physical Mechanism Analysis

To intuitively demonstrate the spatiotemporal feature capture capability of the proposed model, we conducted a comprehensive visualization analysis encompassing macro-regional wind fields and micro-local temporal variations. The visualizations utilize the predictions over the entire study area [37.5° N–53.5° N, 95.5° E–127.5 °E] and focus on specific geographical nodes to assess the model’s adaptability to heterogeneous underlying surfaces.

#### 3.4.1. Spatial Wind Field Distribution

**Figure 8:**
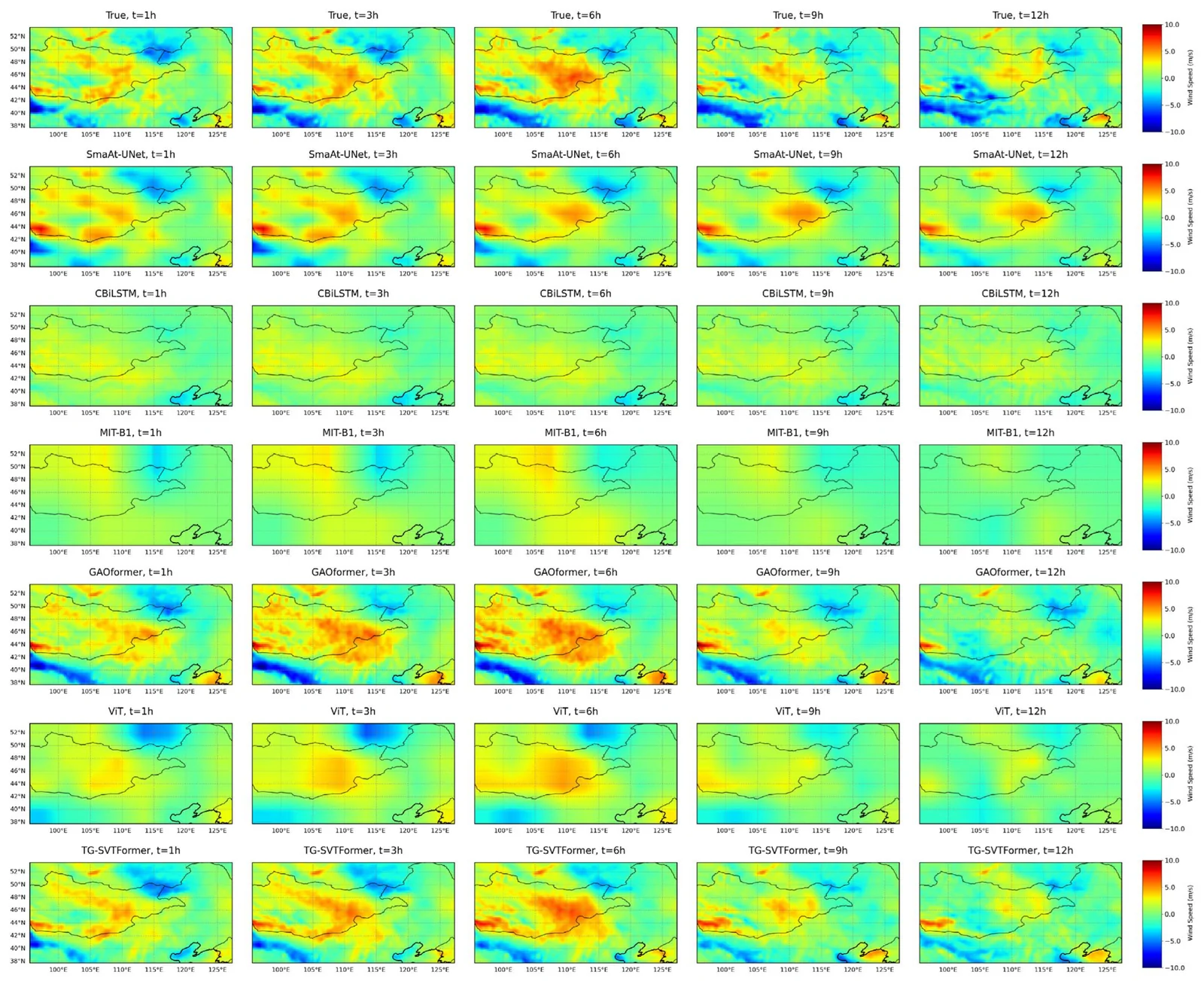
Prediction of the U-wind component at the 1400th test sample.

**Figure 9:**
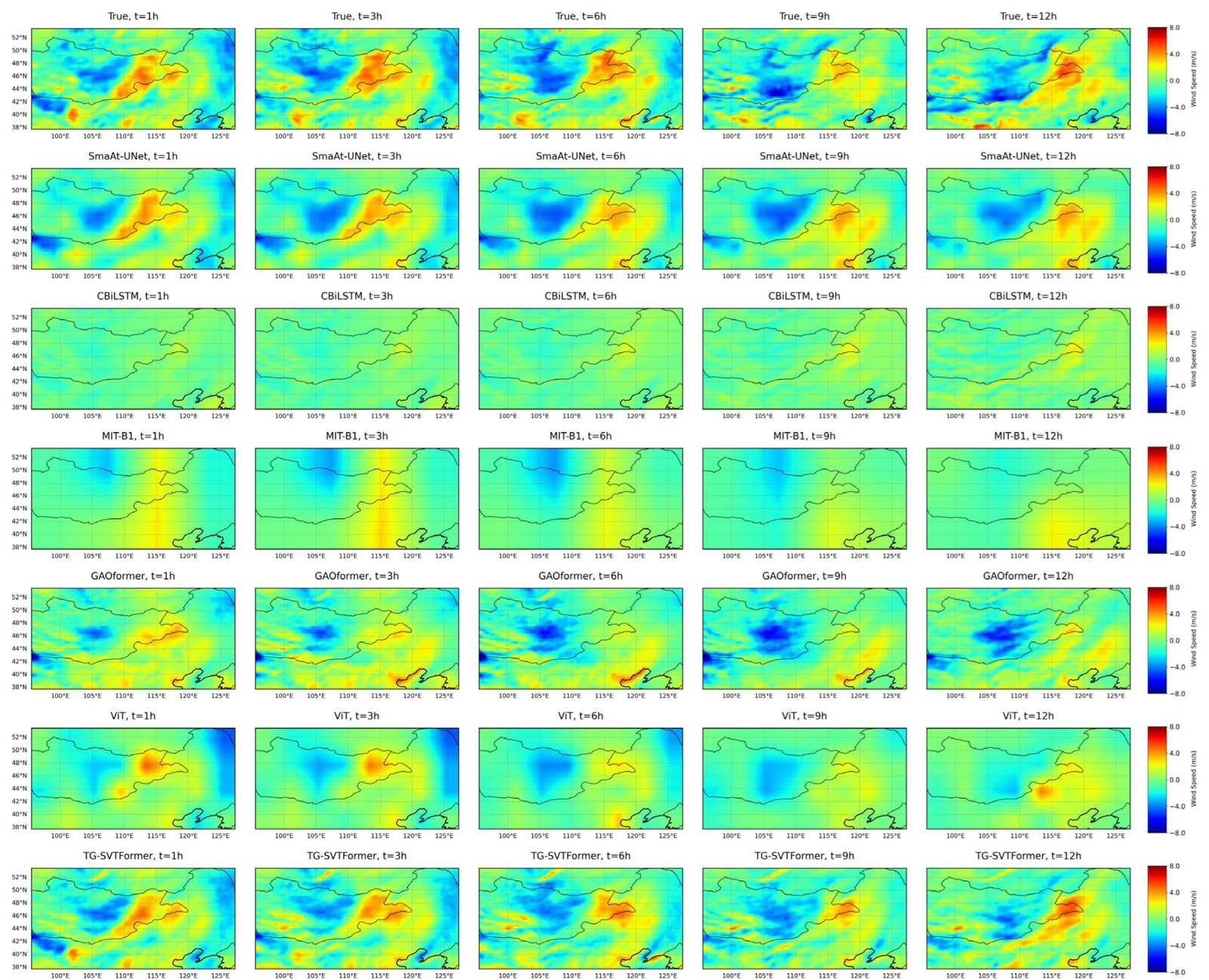
Prediction of the V-wind component at the 1400th test sample.

The wind fields in Northern and Northeastern China exhibit pronounced spatial heterogeneity, driven by the complex topography that induces sharp pressure gradients and localized aerodynamic turbulence. Figure 8 and Figure 9 illustrate the spatial distribution of the predicted U and V wind components against the ERA5 ground truth and baseline models over consecutive forecasting horizons (1 h, 3 h, 6 h, 9 h, and 12 h). Negative values indicate northerly and easterly wind directions, while positive values denote southerly and westerly wind directions. Physically, the baseline models exhibit distinct dynamical degradation as the prediction horizon extends, primarily manifesting as a loss of high-frequency atmospheric energy. Recurrent architectures like CBiLSTM fail to maintain spatial coherence, resulting in fragmented wind patches. This indicates an inability to physically resolve the continuous advection of momentum, leading to unphysical discontinuities in the wind field. While SmaAt-UNet and GAOformer reliably estimate the macroscopic wind speed distribution in the very short term (1–3 h), their predictions become progressively over-smoothed after 6 h. This over-smoothing signifies severe numerical diffusion, where the models artificially dissipate turbulent kinetic energy (TKE) and fail to capture the rapidly shifting, non-linear properties of the boundary layer. This suggests that standard spatial attention or convolutional mechanisms lack the inherent physical inductive bias required to preserve mesoscale eddy structures under long-term temporal accumulation.

In contrast, the proposed TG-SVTFormer maintains a physically consistent spatial structure with the ERA5 truth even at the 12-h horizon. The model accurately preserves sharp spatial gradients and localized high-wind-speed zones while mitigating artificial gradient blurring. This superior physical fidelity is largely attributed to the proposed vertical decoupled temporal hub. By decoupling the temporal and vertical dimensions, the model mitigates the accumulation of spatiotemporal errors, effectively acting as a physics-informed constraint that preserves the vertical structure of the atmospheric boundary layer. Equally important is the dual-stage topography-guided mechanism embedded within both the encoder and decoder. By injecting physical terrain constraints into the feature learning process, this mechanism empowers the network to physically perceive boundary layer aerodynamic friction and terrain-induced wind shear. Consequently, TG-SVTFormer avoids the numerical diffusion plaguing other models, providing a dynamically robust reconstruction of the complex wind field.

#### 3.4.2. Analysis of Diverse Topographical Nodes

The underlying surface characteristics dictate the local momentum and thermal budgets, fundamentally altering regional wind regimes. To evaluate the model’s predictive robustness across varying geographical constraints, two highly representative locations were selected: Location 1 (Inner Mongolia Plateau, 43.5° N, 112° E) and Location 2 (Hexigten Banner, 43.5° N, 117.5° E).

Location 1 represents a typical vast, undulating high plain with an arid/semi-arid climate. The flat terrain minimizes aerodynamic drag, allowing the wind field to be primarily governed by large-scale synoptic forcings and unobstructed sweeping airflows. Consequently, the wind regime here exhibits strong temporal persistence and high-momentum characteristics, particularly during spring and winter. In contrast, Location 2 is situated at the transitional zone between the southern foothills of the Greater Khingan Mountains and the plateau. This complex topography—comprising mountains, intersecting ravines, and forests—induces significant aerodynamic drag, wind shear, and orographic lifting. These mechanisms generate localized mesoscale circulations (e.g., mountain-valley breezes) and form a localized “rain island.” As a result, the wind vector field at Location 2 is highly non-stationary, characterized by high-frequency fluctuations and abrupt wind shifts, presenting a severe challenge for time-series forecasting.

**Figure 10:**
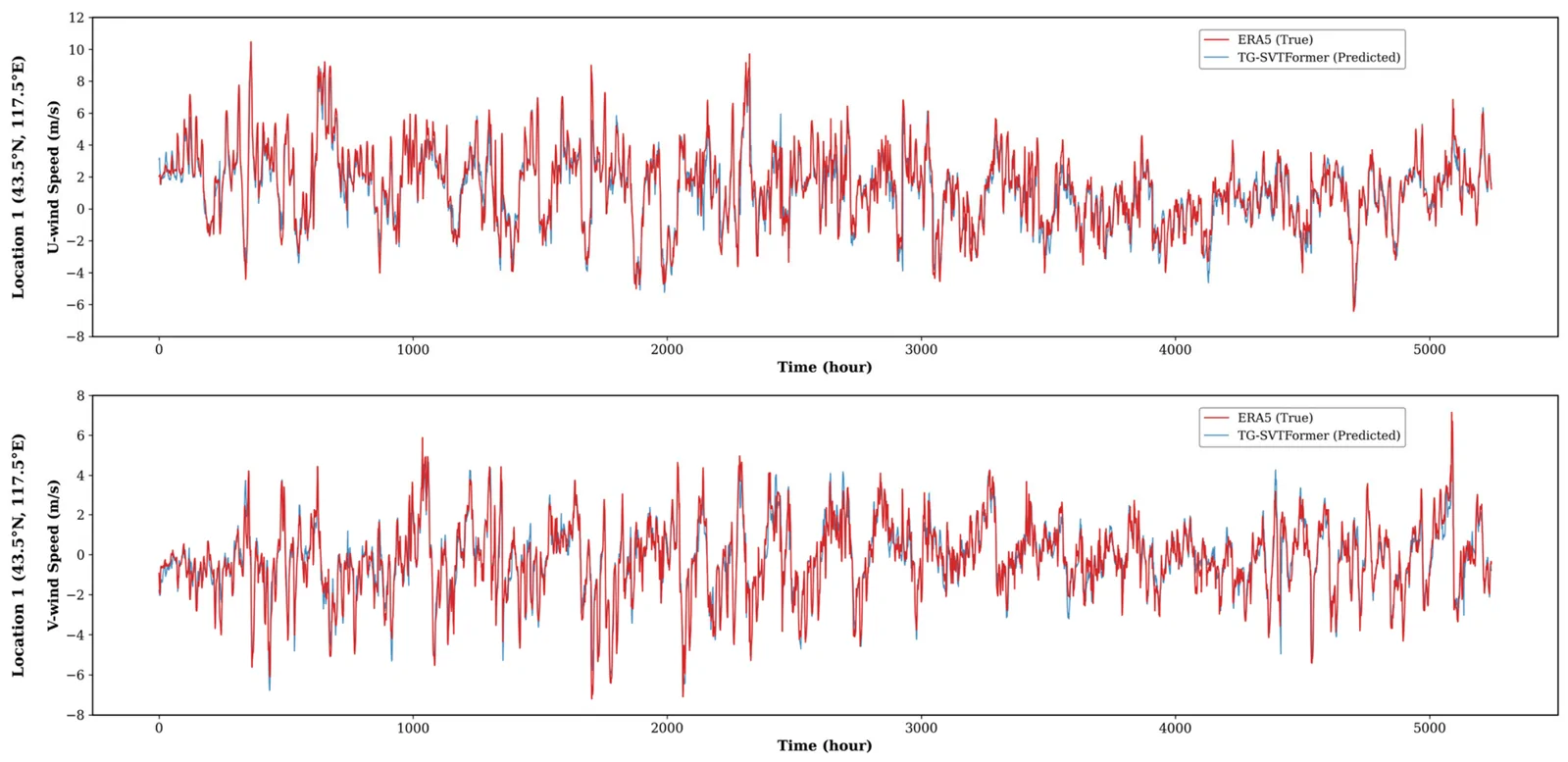
Comparison of wind speed predictions at location 1 (Inner Mongolia Plateau, 43.5° N, 112° E).

**Figure 11:**
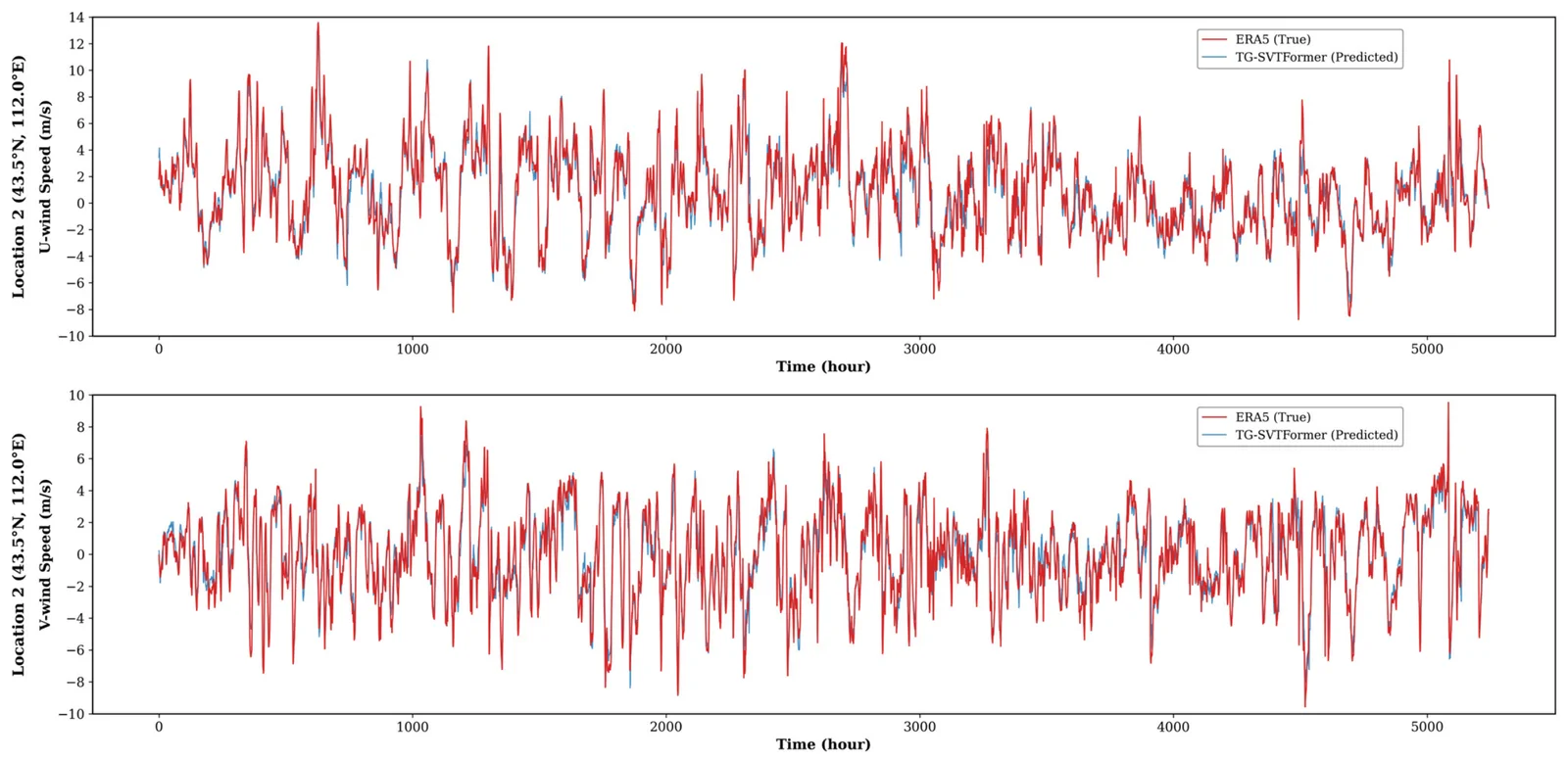
Comparison of wind speed predictions at location 2 (Hexigten Banner, 43.5° N, 117.5° E).

Figure 10 and Figure 11 illustrate the 12-h prediction results (using the first 3 h) across an entire year for both locations. Physically, the time-series plots reveal the model’s ability to resolve distinct atmospheric boundary layer processes. For Location 1, the TG-SVTFormer accurately reproduces the high-momentum wind trends and the sustained U/V wind speeds. This indicates that the model successfully captures the large-scale synoptic forcing and the lack of surface friction typical of a flat plateau, maintaining phase alignment during prolonged high-wind events.

More critically, for the topographically complex Location 2, the TG-SVTFormer demonstrates superior capability in capturing rapid fluctuations at peak and valley moments. The physical significance here lies in the model’s implicit handling of sub-grid orographic drag and non-stationary signals. The sharp peaks and sudden drops in wind speed are physical manifestations of mountain-wave breaking, gap flows (venturi effects) within ravines, and boundary layer separation. The close alignment with ERA5 ground truth suggests that TG-SVTFormer can effectively learn the complex, nonlinear dynamic responses of the atmosphere to rugged terrain, overcoming the tendency of traditional models to over-smooth extreme values in heterogeneous landscapes. This confirms the model’s physical robustness in resolving both synoptic-scale continuity and mesoscale topographic forcing.

#### 3.4.3. Analysis of Wind Direction Prediction

**Figure 12:**
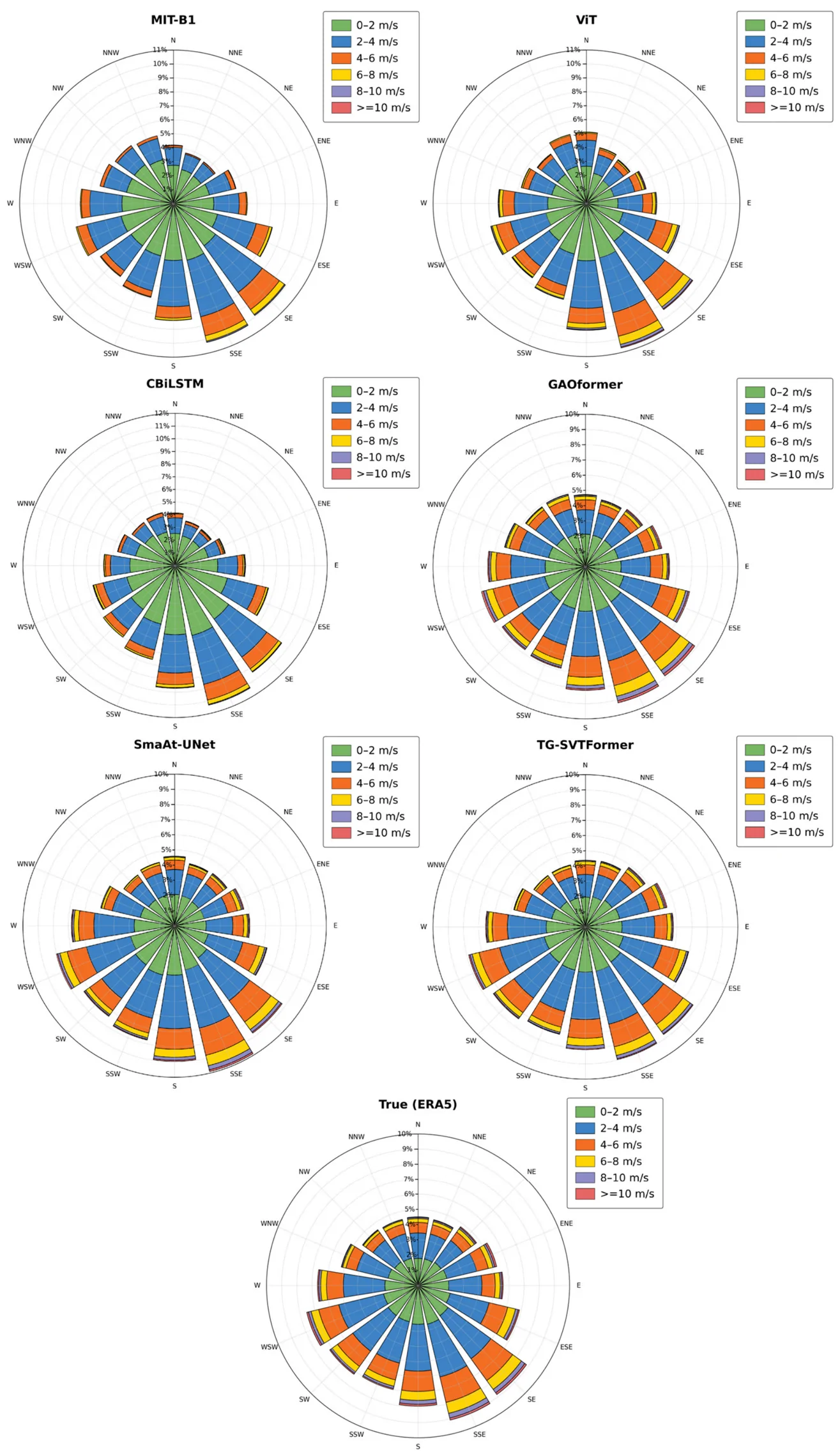
The comparative wind rose diagrams of the different forecasting models.

Figure 12 presents the wind rose diagrams, which characterize the climatological wind regime of the study area. From a physical and meteorological perspective, the wind rose reveals a highly asymmetric and skewed directional distribution, reflecting the combined effects of large-scale synoptic circulation and local topographical channeling. The region is predominantly influenced by winds from the southeast to south, with south-southwesterly to west-southwesterly winds accounting for a non-negligible proportion. This directional preference is physically linked to the regional atmospheric circulation patterns (e.g., the East Asian monsoon system and the passage of extratropical cyclones), where the interaction between the prevailing pressure gradients and the complex terrain of the Inner Mongolia Plateau and Greater Khingan Mountains creates preferred corridors for airflow.

Notably, the physical coupling between wind direction and momentum transport is evident, as high-speed wind events (exceeding 10 m/s) are heavily clustered within these dominant directional corridors. These high-momentum events are typically associated with strong cold air outbreaks (e.g., Siberian High intrusions) that are dynamically channeled through these specific geographical gaps, leading to localized wind acceleration due to the venturi effect.

The physical significance of the model’s performance lies in its ability to resolve this non-stationary, directionally dependent momentum distribution. As observed in Figure 12, while baseline models (e.g., CBiLSTM, GAOformer) exhibit a tendency to over-smooth the directional extremes—a common pitfall of standard attention mechanisms that treat directional variables as continuous linear scalars—the proposed TG-SVTFormer effectively captures the sharp frequency peaks and the high-speed wind clusters within the dominant sectors. This indicates that TG-SVTFormer successfully addresses the 360-degree periodic nature of directional variables. By preserving the physical boundaries between directional sectors, the model mitigates the over-smoothing tendency and accurately reconstructs the skewed climatological wind distribution, demonstrating superior physical consistency with ERA5 observations.

Overall, by integrating topography guidance and decoupling the temporal–vertical dependencies, the proposed model mitigates the over-smoothing of high-frequency atmospheric dynamics, providing an effective framework for wind vector forecasting.

## 4. Conclusions

Regional wind speed vector forecasting is critical for ensuring the safe operation and stable integration of wind power into modern smart grids. However, capturing the complex, multi-scale atmospheric dynamics over intricate terrains remains a significant challenge. To address this issue, this study proposes a topography-integrated and decoupled spatio-vertical–temporal deep learning architecture, specifically tailored for regional multi-step WSVF. Extensive experiments conducted on multi-pressure-level reanalysis datasets fully validate the effectiveness of the proposed model. More importantly, the findings of this study offer a new perspective on how physically informed decomposition and terrain-aware representation learning can jointly advance the predictability of complex atmospheric systems, rather than merely improving a single forecasting metric.

The main conclusions of this study are summarized as follows: (a) Dismantling the traditional spatiotemporal entanglement significantly improves multi-step forecasting performance. By decoupling the vertical dimension and isolating spatial representations across individual pressure levels, TG-SVTFormer effectively mitigates cumulative error propagation. This finding is significant because it suggests that the long-standing performance bottleneck in multi-step wind vector forecasting may stem not only from limited model capacity, but also from an inappropriate entanglement of physical dimensions. (b) Incorporating static topographical variables provides important physical constraints for the network. This topography-guided strategy enables the model to effectively capture aerodynamic friction and terrain-induced wind shear, enhancing its capacity to model complex meteorological systems. The significance of this result lies in demonstrating that static geographic priors are not merely auxiliary inputs, but act as meaningful physical regularizers that embed terrain-aware dynamics into the learning process. This bridges the gap between purely data-driven architectures and terrain-sensitive atmospheric physics, offering a practical pathway for improving forecasting skill in regions where topographic forcing is a first-order control on wind behavior. (c) Customizing the MiT-B1 backbone proves effective. By removing conventional downsampling in deep stages and incorporating dilated convolutions, the model broadens the global receptive field while maintaining fine-grained spatial details. This result highlights a broader architectural insight: for regional atmospheric forecasting, preserving fine-scale spatial information while expanding contextual awareness is more valuable than blindly deepening or downsampling a backbone. It suggests that future geophysical Transformer designs should prioritize resolution-preserving receptive field expansion, which is essential for resolving local wind structures without sacrificing large-scale meteorological context. (d) The integration of high-level atmospheric wind vectors to mimic vertical momentum transport is empirically shown to stabilize long-horizon predictions. This physics-guided constraint acts as a reliable anchor against chaotic meteorological volatility, helping to avoid the degradation of directional accuracy observed in traditional purely data-driven models. This finding is particularly significant because it shows that embedding a simplified representation of vertical momentum transport can effectively constrain chaotic error growth in long-horizon forecasting. It offers empirical evidence that physics-guided constraints, even when approximate, can serve as stability anchors in deep learning models, thereby improving the operational credibility of data-driven wind vector forecasting for power system decision-making.

Although the architecture of TG-SVTFormer is designed to achieve multi-step forecasting of wind vectors over intricate topography, its generalization capability across diverse climatic regimes and extended temporal scales requires further empirical verification. Future work will focus on integrating multi-source observations—including real-time Doppler radar, satellite imagery, high-resolution radiosonde profiles, and ground-based remote sensing—to further enhance the model’s ability to capture extreme meteorological events. Furthermore, given the inherent computational complexity of hierarchical Transformers, investigating lightweight network optimization paradigms will be crucial. These optimizations aim to reduce computational overhead and accelerate inference speeds, thereby facilitating practical deployment on edge devices for real-time wind farm control and microgrid dispatching. Ultimately, this study contributes not only a high-performance forecasting architecture, but also a set of physics-informed design principles for spatio-temporal geophysical modeling. These principles may help shift the community from treating deep forecasting models as opaque black boxes toward building physically structured, terrain-aware, and operationally trustworthy forecasting systems for renewable energy integration.

## Figures and Tables

**Table 1:** Model Parameters and Channel Dimensions for the MiT Encoder Series.

Model Variant	MiT-B0	MiT-B1	MiT-B2	MiT-B3	MiT-B4	MiT-B5
Encoder Params (M)	~3.4	~13.1	~24.2	~44.0	~60.8	~81.4
Channel Dimensions (C1, C2, C3, C4)	(32, 64, 160, 256)	(64, 128, 320, 512)	(64, 128, 320, 512)	(64, 128, 320, 512)	(64, 128, 320, 512)	(64, 128, 320, 512)

**Table 2:** Ablation study on the MAE performance of different TG-SVTFormer variants for WSVF over the next 12 h.

Model	V-Wind [m/s]						U-Wind [m/s]					
	1 h	2 h	3 h	6 h	9 h	12 h	1 h	2 h	3 h	6 h	9 h	12 h
C1	0.68	0.74	0.78	0.90	1.00	1.09	0.67	0.72	0.76	0.89	0.98	1.08
C2	1.19	1.11	1.05	1.02	1.21	1.49	1.11	1.04	1.00	1.00	1.18	1.43
C3	0.92	0.97	1.02	1.15	1.24	1.32	0.89	0.94	0.99	1.12	1.20	1.29
C4	0.57	0.65	0.71	0.85	0.95	1.06	0.56	0.64	0.69	0.84	0.94	1.04

**Table 3:** Ablation study on the RMSE performance of different TG-SVTFormer variants for WSVF over the next 12 h.

Model	V-Wind [m/s]						U-Wind [m/s]					
	1 h	2 h	3 h	6 h	9 h	12 h	1 h	2 h	3 h	6 h	9 h	12 h
C1	1.01	1.08	1.14	1.34	1.49	1.62	0.98	1.06	1.12	1.31	1.46	1.60
C2	1.65	1.54	1.46	1.45	1.72	2.09	1.53	1.44	1.38	1.42	1.68	2.01
C3	1.30	1.36	1.42	1.61	1.73	1.84	1.26	1.33	1.39	1.57	1.70	1.81
C4	0.85	0.95	1.04	1.27	1.42	1.57	0.82	0.93	1.02	1.25	1.41	1.55

**Table 4:** Ablation study on the ACC performance of different TG-SVTFormer variants for WSVF over the next 12 h.

Model	V-Wind						U-Wind					
	1 h	2 h	3 h	6 h	9 h	12 h	1 h	2 h	3 h	6 h	9 h	12 h
C1	0.93	0.91	0.90	0.87	0.84	0.81	0.92	0.91	0.89	0.86	0.82	0.78
C2	0.79	0.82	0.84	0.84	0.77	0.66	0.81	0.83	0.84	0.83	0.76	0.65
C3	0.87	0.86	0.84	0.80	0.77	0.73	0.86	0.85	0.83	0.78	0.74	0.70
C4	0.95	0.93	0.92	0.88	0.85	0.82	0.95	0.93	0.92	0.87	0.84	0.80

**Table 5:** Ablation study on the WDFA performance of different TG-SVTFormer variants for WSVF over the next 12 h.

Model	1 h (%)			3 h (%)			6 h (%)			12 h (%)		
	α = 90	α = 45	α = 22.5	α = 90	α = 45	α = 22.5	α = 90	α = 45	α = 22.5	α = 90	α = 45	α = 22.5
C1	96.19	89.18	74.67	95.19	86.64	70.08	93.62	83.15	64.70	90.60	76.97	56.31
C2	89.97	74.49	52.14	92.14	79.01	57.57	92.25	79.59	58.66	83.41	63.64	41.22
C3	93.70	82.09	62.68	92.37	79.00	57.95	90.22	74.62	52.22	87.01	68.73	45.57
C4	97.38	92.24	80.16	95.99	88.65	73.42	94.10	84.36	66.71	90.90	77.92	57.88

**Table 6:** MAE results of different models for WSVF over the next 12 h.

Model	V-Wind [m/s]						U-Wind [m/s]					
	1 h	2 h	3 h	6 h	9 h	12 h	1 h	2 h	3 h	6 h	9 h	12 h
ViT	1.11	1.13	1.15	1.24	1.31	1.38	1.09	1.12	1.14	1.22	1.29	1.36
MiT-B1	1.51	1.52	1.52	1.56	1.60	1.64	1.47	1.48	1.49	1.53	1.57	1.61
CBiLSTM	1.53	1.51	1.49	1.47	1.53	1.60	1.49	1.47	1.46	1.45	1.50	1.57
GAOformer	1.12	1.13	1.14	1.21	1.30	1.39	1.09	1.10	1.11	1.20	1.28	1.38
SmaAt-UNet	0.78	0.84	0.91	1.13	1.25	1.40	0.76	0.80	0.87	1.09	1.22	1.39
TG-SVTFormer	0.57	0.65	0.71	0.85	0.95	1.06	0.56	0.64	0.69	0.84	0.94	1.04

**Table 7:** RMSE results of different models for WSVF over the next 12 h.

Model	V-Wind [m/s]						U-Wind [m/s]					
	1 h	2 h	3 h	6 h	9 h	12 h	1 h	2 h	3 h	6 h	9 h	12 h
ViT	1.52	1.55	1.58	1.70	1.81	1.90	1.51	1.54	1.57	1.68	1.79	1.88
MiT-B1	2.04	2.05	2.06	2.11	2.16	2.21	1.99	2.00	2.02	2.07	2.13	2.19
CBiLSTM	2.08	2.05	2.04	2.03	2.09	2.18	2.01	1.99	1.98	1.98	2.05	2.15
GAOformer	1.57	1.58	1.60	1.72	1.84	1.98	1.52	1.53	1.56	1.69	1.82	1.96
SmaAt-UNet	1.08	1.16	1.26	1.55	1.74	1.93	1.05	1.10	1.19	1.51	1.69	1.92
TG-SVTFormer	0.85	0.95	1.04	1.27	1.42	1.57	0.82	0.93	1.02	1.25	1.41	1.55

**Table 8:** ACC results of different models for WSVF over the next 12 h.

Model	V-Wind						U-Wind					
	1 h	2 h	3 h	6 h	9 h	12 h	1 h	2 h	3 h	6 h	9 h	12 h
ViT	0.82	0.81	0.80	0.77	0.74	0.71	0.79	0.78	0.77	0.74	0.70	0.66
MiT-B1	0.63	0.63	0.63	0.61	0.59	0.56	0.59	0.59	0.58	0.56	0.53	0.50
CBiLSTM	0.64	0.65	0.66	0.66	0.63	0.59	0.62	0.62	0.63	0.62	0.60	0.55
GAOformer	0.82	0.82	0.82	0.79	0.76	0.72	0.82	0.81	0.81	0.77	0.74	0.69
SmaAt-UNet	0.91	0.90	0.88	0.82	0.77	0.71	0.91	0.90	0.88	0.81	0.75	0.68
TG-SVTFormer	0.95	0.93	0.92	0.88	0.85	0.82	0.95	0.93	0.92	0.87	0.84	0.80

**Table 9:** WDFA results of different models for WSVF over the next 12 h.

Model	1 h (%)			3 h (%)			6 h (%)			12 h (%)		
	α = 90	α = 45	α = 22.5	α = 90	α = 45	α = 22.5	α = 90	α = 45	α = 22.5	α = 90	α = 45	α = 22.5
ViT	91.20	76.43	54.83	90.48	74.88	52.67	89.09	72.14	49.29	86.07	66.70	43.20
MiT-B1	85.00	64.11	40.53	84.54	63.28	39.73	83.51	61.65	38.09	81.10	57.90	34.79
CBiLSTM	81.49	59.84	37.09	82.43	61.34	38.43	82.77	61.93	38.98	79.21	56.58	34.33
GAOformer	91.31	77.37	55.46	90.97	76.63	54.48	89.75	74.26	51.63	86.72	68.63	45.49
SmaAt-UNet	95.44	86.32	68.78	93.91	82.46	62.40	90.74	75.28	52.46	85.25	65.38	41.80
TG-SVTFormer	97.38	92.24	80.16	95.99	88.65	73.42	94.10	84.36	66.71	90.90	77.92	57.88

## Data Availability

The datasets supporting the findings of this study are available as follows: (1) Wind data: The wind data are obtainable from the European Center for Medium-Range Weather Forecasting (https://cds.climate.copernicus.eu/ (accessed on 28 September 2026)); (2) Elevation data: The elevation data are obtainable from the National Oceanic and Atmospheric Administration (https://www.ncei.noaa.gov/ (accessed on 28 September 2026)). All data access dates were on 30 April 2026.

## Abbreviations

The following abbreviations are used in this manuscript:

ACC	Anomaly Correlation Coefficient
AI	Artificial Intelligence
CAM	Channel Attention Mechanism
CNN	Convolutional Neural Network
ECMWF	European Centre for Medium-Range Weather Forecasts
GELU	Gaussian Error Linear Unit
GRU	Gated Recurrent Unit
INN	Invertible Neural Network
LSTM	Long Short-Term Memory
MAE	Mean Absolute Error
MiT	Mix-Transformer
MiT-B1	Mix-Transformer B1 variant
MLP	Multilayer Perceptron
MSE	Mean Squared Error
OPE	Overlapping Patch Embedding
ReLU	Rectified Linear Unit
RMSE	Root Mean Square Error
RNN	Recurrent Neural Network
SSIM	Structural Similarity
TG-SVTFormer	Topography-Guided Spatio-Vertical–Temporal Transformer
U-Net	U-shaped Network
ViT	Vision Transformer
WDFA	Wind Direction Forecasting Accuracy
WRF	Weather Research and Forecasting
WSF	Wind Speed Forecasting
WSVF	Wind Speed Vector Forecasting
